# Glucagon and Glucose Availability Influence Metabolic Heterogeneity and Malignancy in Pancreatic Neuroendocrine Tumour (pNET) Cells: Novel Routes for Therapeutic Targeting

**DOI:** 10.3390/molecules30132736

**Published:** 2025-06-25

**Authors:** Bárbara Ferreira, Isabel Lemos, Cindy Mendes, Beatriz Chumbinho, Fernanda Silva, Daniela Pereira, Emanuel Vigia, Luís G. Gonçalves, António Figueiredo, Daniela Cavaco, Jacinta Serpa

**Affiliations:** 1 iNOVA4Health, NOVA Medical School, Campo dos Mártires da Pátria, 130, 1169-056 Lisboa, Portugal; a2021084@nms.unl.pt (B.F.); a2022507@nms.unl.pt (I.L.); cindy.mendes@nms.unl.pt (C.M.); fernanda.silva@nms.unl.pt (F.S.); emanuel.duarte@chlc.min-saude.pt (E.V.); 2 Portuguese Institute of Oncology of Lisbon Francisco Gentil (IPOLFG), Rua Prof Lima Basto, 1099-023 Lisboa, Portugal; dcpereira@ipolisboa.min-saude.pt (D.P.); daniela.rcavaco@gmail.com (D.C.); 3 Universitary Center and Hospital of Central Lisbon, Hospital Curry Cabral, Rua da Beneficência, 1069-166 Lisboa, Portugal; beatriz.chumbinho@gmail.com (B.C.); antonio.figueiredo@chlc.min-saude.pt (A.F.); 4 Institute of Chemical and Biological Tecnology António Xavier (ITQB NOVA), Avenida da República (EAN), 2780-157 Oeiras, Portugal; lgafeira@itqb.unl.pt

**Keywords:** glucagon, pancreatic neuroendocrine tumours (pNETs), cancer metabolism, biomarkers, glucagon receptor (GCGR), glucagon-like peptide-1 receptor (GLP-1R)

## Abstract

Cancer metabolism is a hallmark of cancer. However, the impact of systemic metabolism and diet on tumour evolution is less understood. This study delves into the role of glucagon, as a component of the pancreatic microenvironment, in regulating features of pancreatic neuroendocrine tumour (pNET) cells and the metabolic remodelling occurring in the presence and absence of glucose. pNET cell lines (BON-1 and QGP-1) and the non-malignant pancreatic α-TC1 cell line were used as models. Results showed that pNET cells responded differently to glucose deprivation than α-TC1 cells. Specifically, pNET cells upregulated the GCGR in the absence of glucose, while α-TC1 cells did so in high-glucose conditions, allowing the glucagon-related pERK1/2 activation under these conditions in pNET cells. Glucagon enhanced cancerous features in pNET BON-1 cells under glucose-deprived and hyperglucagonemia-compatible concentrations. In the α-TC1 cell line, glucagon modulated cell features under high-glucose and physiological glucagon levels. NMR exometabolome analysis revealed differences in metabolic processes based on glucose availability and glucagon stimulation across cell lines, highlighting amino acid metabolism, glycolysis, and gluconeogenesis. The expression of metabolic genes was consistent with these findings. Interestingly, QGP-1 and α-TC1 cells produced glucose in no-glucose conditions, and glucagon upregulated glucose production in α-TC1 cells. This suggests that gluconeogenesis may be beneficial for some pNET subsets, pointing out novel metabolism-based strategies to manage pNETs, as well as a step forward in endocrinology and systemic metabolism. The association between GCGR expression and malignancy and a negative correlation between glucagon receptor (GCGR) and glucagon-like peptide-1 receptor (GLP-1R) expression was observed, indicating a biological role of glucagon in pNETs that deserves to be explored.

## 1. Introduction

Metabolic remodelling is a requirement for proliferating cancer cells, and it has received increased focus during the last decade [1]. Cancer cells, in general, have demonstrated enhanced consumption of glucose and lactate production even under normoxic circumstances [2,3,4,5,6]. The increased glucose consumption rate is undoubtedly a metabolic hallmark of cancer cells; however, oxidative phosphorylation (OXPHOS) occurs concomitantly with glycolysis using substrates other than glucose [7]. On the other hand, glycolysis is primarily a precursor-supplying route for different metabolic biosynthetic pathways [7]. Thus, cancer cells use intermediates of the glycolytic pathway as precursors for synthesizing amino acids, nucleic acids, and lipids, as well as to sustain the tumour cell’s antioxidant defences against hostile tumour microenvironments (TMEs) and chemotherapeutic agents [8].

The impact of systemic metabolism and diet on tumour evolution is still not fully comprehended, necessitating new experiments exploring the influence of systemic metabolic controllers such as insulin and glucagon. The effect of insulin has been rather exploited in cancer, but glucagon has been passed over. Glucagon is a peptide hormone produced by the α-cells of the pancreatic Langerhans islets upon hypoglycaemia [9] and exerts its effects by binding to the glucagon receptor (GCGR), a G-protein coupled receptor (GPCR) expressed primarily in liver cells [10] and certain cancer cell types, including pancreatic neuroendocrine tumours (pNETs) [11]. Upon binding, the GCGR activates intracellular signalling pathways such as the second messenger cyclic AMP-dependent protein kinase A (cAMP-PKA) pathway, which triggers glycogenolysis and gluconeogenesis in hepatocytes to increase blood glucose levels [10,12,13].

In cancer cells, particularly pNETs, GCGR activation may also support malignant features by upregulating pro-survival and proliferation pathways, such as mitogen-activated protein kinase/extracellular signal-regulated kinase (MAPK/ERK), commonly associated with cell proliferation and survival, especially under metabolic stress conditions [14,15].

Therefore, the role of glucagon in human glucose homeostasis is unveiled, although at the cellular level, especially in cancer cells, much information is yet to be explored. Although insulin’s impact has been addressed in different cancer models, the impact of glucagon and hyperglucagonemia (>100 pmol/mL) on the TME and consequent effect on cancer metabolic remodelling is not well understood.

pNETs are a subtype of neuroendocrine neoplasms constituting 1–2% of all pancreatic neoplasms [16,17,18], and they are derived from the islet of Langerhans cells with various morphologies and behaviours, including metastatic potential [19]. The incidence of pNETs is rising in the western world [20], and these tumours are divided into functioning and non-functioning types, ranging from indolent to aggressive, depending on histology and the production of active hormones [21], including glucagon [22]. The majority of pNETs (about 60%) are non-functioning [22], and patients present obscure symptoms or are asymptomatic. Therefore, about 80% of pNETs are metastatic at diagnosis and the 5-year survival rate is 40–60% [16,23]. In addition, some studies in patients and animal models indicate pancreatic α-cell hyperplasia (ACH) as an important differential diagnosis of hyperglucagonemia upon inactivating mutations in the GCGR [11,24,25,26]. Furthermore, ACH is considered a preneoplastic lesion in the stepwise trajectory of pNET carcinogenesis [25,27]. This evidence reinforces the need for more studies to understand pNET biology further.

In addition to the GCGR, the glucagon-like peptide-1 receptor (GLP-1R) plays a role in modulating glucagon’s effects within the pancreatic microenvironment, being primarily known for inhibiting glucagon exocytosis by α-cells [28]. Thus, the binding of glucagon to the glucagon-like peptide-1 receptor (GLP-1R) may subvert the system and contribute to hyperglucagonemia and the activation of signalling that may favour cancer. The subversive loop is influenced by glucagon levels in the tumour and organ microenvironment (TOME), as well as the mutational profile and expression levels of the GCGR and GLP-1R [29].

In this study, the main goal is to investigate the role of glucagon as a component of the pancreatic microenvironment in the metabolic remodelling of pNETs, given that glucagon’s influence on cellular signalling pathways could be a driving factor in pNET malignancy. Considering that all pancreatic pNETs arise in the Langerhans islets, whose microenvironment is glucagon-rich [30], and understanding how glucagon promotes tumour survival and metabolic remodelling in pNETs may provide insights into new therapeutic targets.

Specifically, we aim to explore the impact of glucagon on pNET cell features, depending on glucose bioavailability. Additionally, we attempt to explore the potential utility of the GCGR and GLP-1R (the receptor of GLP-1, which is the repressor of glucagon exocytosis by α-cells) [12,13], as prognosis markers and/or therapeutic targets. The role of glucagon as a cancer regulator, and the usefulness of the GCGR and GLP1-R as biomarkers are underexplored.

## 2. Materials and Methods

### 2.1. Cell Culture

Two pancreatic neuroendocrine tumour (pNET) cell lines, BON-1 (CVCL-3985; JCRB cell Bank, Ibaraki City, Osaka, Japan), derived from a lymph node metastasis of a pancreatic serotonin-producing carcinoid [31], and QGP-1 (CVCL-3143; JCRB cell Bank), derived from a somatostatinoma [32], were used in this study. Additionally, a non-malignant immortalized α-cell line from pancreatic islets of Langerhans, α -TC1 clone 6 (CRL-2934™, ATCC, Manassas, VA, USA), was used as a control for glucagon stimulus on cell features and metabolism, since glucagon’s function in mice and humans is conserved [33,34]. BON-1 cells were cultured in Dulbecco’s modified Eagle medium/nutrient mixture F-12 (Ham) 1× (DMEM/F-12) (11330-032, Gibco, Life Technologies, Waltham, MA, USA), while QGP-1 cells were cultured in Roswell Park Memorial Institute (RPMI) 1640 Medium 1× (12-167F, Lonza, Bioscience, Basel, Switzerland) supplemented with L-glutamine. α-TC1 clone 6 cells were cultured in Dulbecco’s modified Eagle medium 1× (DMEM) (41965-039, Gibco, Life Technologies, MA, USA) containing 4.5 g/L of D-glucose and 0.58 g/L L-glutamine. No-glucose experiments were performed using DMEM (P04-01549, PAN Biotec, Aidenbach, Germany). All cell culture media were supplemented with 10% foetal bovine serum (FBS; S 0615, Merck, Rahway, NJ, USA), 1% antibiotic-–antimycotic (AA; P06-07300, PAN Biotech) and 50 µg/mL gentamicin (15750-060, Gibco, Life Technologies, MA, USA). Cells were plated on gelatin-coated T75 flasks (75 cm^2^) and cultured until they reached 75–100% optical confluency. The detachment of cells was achieved using 0.05% trypsin-EDTA 1× (25300-054, Invitrogen, Waltham, MA, USA) at room temperature (RT), for 3–5 minutes (min). Cells were maintained under 37 °C, 5% CO_2_, in a humidified environment. The concentrations of glucagon (G1774, Sigma-Aldrich, St. Louis, MO, USA) were selected in order to simulate physiological serum concentrations (50 and 100 pg/mL) and hyperglucagonemia, at concentrations compatible with glucagonoma syndrome (250 and 500 pg/mL) [35]. For each assay, the cell number was determined in a Bürker counting chamber. Before any in vitro experiment, cells were synchronized by overnight starvation in 1% FBS culture medium.

### 2.2. Cell Viability

For cell viability, cells were plated in 96-well plates, and depending on the cell line, different numbers of cells were used: BON-1 and QGP-1 cell lines (6 × 10^3^ cells/well/100 µL), and α-TC1 clone 6 (2 × 10^4^ cells/well/100 µL). After starvation, cells were cultured in experimental conditions for 24 and 48 h. Cell viability was assessed using Cell Counting Kit-8 (CCK-8; Dojindo Molecular Technologies, Gaithersburg, MD, USA), according to the manufacturer’s instructions, to evaluate the effect of different concentrations of glucagon (50, 100, 250, and 500 pg/mL; G1774, Sigma-Aldrich, St. Louis, MO, USA) on malignant features of pNET cells and non-malignant α-TC1 cells. Briefly, after treatment conditions, the CCK-8 solution was added to the culture medium and incubated at 37 °C for 2 h. The absorbance related to the conversion of the tetrazolium salt (WST-8) into a water-soluble formazan dye was measured at 450 nm with gentle shaking in an iMark Microplate Absorbance Reader (1681130, Bio-Rad, Hercules, CA, USA). Cell viability was determined using the formula (experimental group absorbance value/control group absorbance value) × 100%, and presented as a fold change to the control.

### 2.3. Proliferation Curves

For cell proliferation assays, cells were plated in 96-well plates, and depending on the cell line, different numbers of cells were used: BON-1 and QGP-1 cell lines (6 × 10^3^ cells/well/100 µL), and α-TC1 clone 6 (2 × 10^4^ cells/well/100 µL). After starvation, cells were cultured in experimental conditions for 24 and 48 h. Cells in suspension were collected to an Eppendorf and adherent cells were washed with PBS (1×), and harvested using 200 µL of 0.05% trypsin-EDTA; then, cells were added to the respective supernatant (conditioned culture media) and centrifuged at 150× *g* for 3 min. The total cell number *per* mL was calculated at different time points (0, 24 and 48 h) using a Neubauer counting chamber.

### 2.4. Immunofluorescence

For the immunofluorescence assay to detect and evaluate the expression of the glucagon receptor (GCGR) and GLP-1 receptor (GLP-1R), cells (1 × 10^5^ cells/ well/500 µL) were seeded on a 24-well plate on the top of coverslips coated with 0.2% gelatin (G-1890, Sigma Aldrich). After 24 h of experimental conditions, cells were fixed with 200 µL of 2% paraformaldehyde for 15 min at RT. Then, cells were rinsed with PBS (1×) and incubated with 50 mM of NH4Cl in PBS (1×). First, 0.5% BSA-0.1% saponin-PBS (*w*/*v*/*v*) was used for 15 min at room temperature (RT) to block and permeabilize cells. Cells were then incubated with primary antibodies (rabbit anti-human GCGR, ab75240, Abcam; rabbit anti-human GLP-1R, 1R1J8, Invitrogen), diluted 1:500 and 1:300, respectively, in 0.5% BSA0.1% saponin-PBS (*w*/*v*/*v*) overnight, at 4 °C with slow agitation. Cells were washed 3 × with 0.5% BSA-0.1% saponin-PBS (*w*/*v*/*v*) and incubated with Alexa Fluor^®^ 488 goat anti-rabbit (A11034, Invitrogen) secondary antibody, diluted 1: 1000 in 0.5% BSA-0.1% saponin-PBS (*w*/*v*/*v*) for 2 h at RT. To validate the specificity of the secondary antibody, negative controls without primary antibodies were used. Cells were washed with 0.5% BSA-0.1% saponin-PBS (*w*/*v*/*v*) and then rinsed with PBS (1×). The coverslips were mounted in slides with VECTASHIELD media with DAPI (4′-6-diamidino-2-phenylindole) (H-1200, VectorLabs). The analysis was performed by standard fluorescence microscopy using a Zeiss Imajer.Z1 AX10 microscope. Images were acquired and processed with CytoVision v.7.1 software and quantified using Image J v.1.53 software (rsb.info.nih.gov/ij/).

### 2.5. Relative Quantifying Real-Time Polymerase Chain Reaction (RQ-PCR)

For gene expression analysis, mRNA was quantified by RT plus RQ-PCR. For this, 2 × 10^5^ cells/wells/1 mL were cultured in 12-well plates. After starvation, cells were cultured under experimental conditions for 48 h. RNA extraction was performed using the RNeasy Mini Kit Qiagen^®^ (74104, Qiagen, Hilden, Germany) according to the manufacturer’s protocol. The concentration of RNA samples was measured at 260 nm using a NanoDrop 2000 (ND-2000, Thermo Scientific, Waltham, MA, USA). For cDNA synthesis, 0.5 µg of total RNA was incubated at 70 °C for 10 min with random primers (11034731001, Roche, Basel, Switzerland) and water to achieve a final volume of 8 µL. Subsequently, cDNA was synthesized using SuperScript™ II reverse transcriptase (18080-44, Invitrogen), RNAse OUT™ (10777-019, Invitrogen), and a mix of deoxynucleotides (dNTPs) (10 mM; 28-4065-22V, 28-4065-02V, 28-4065-12V, and 28-4065-32V, GE Healthcare) following the manufacturer’s protocol. The influence of glucagon on the expression of metabolic key genes was evaluated by RQ-PCR using specific primers for human genes (Table 1) and SYBR^®^ Green Master Mix (04707516001, Roche, Basel, Switzerland) according to the manufacturer’s instructions. The reactions and quantification were conducted using a Lightcycler^®^ 480 System instrument (05015243001, Roche). The hypoxanthine-guanine phosphoribosyltransferase 1 gene (HPRT1) served as a reference or housekeeping gene for normalization purposes.

### 2.6. Wound Healing Assay

For the wound-healing assay, 3.5 × 10^5^ cells/well/1 mL were plated in 12-well plates and cultured onto gelatin until they formed a confluent monolayer. To ensure the accuracy of the wound healing progression, cell proliferation was inhibited by the addition of mitomycin-C, an effective antimitotic agent, (M4287, Sigma), followed by incubation for 3 h. Next, a linear scratch, simulating a wound, was created across the diameter of each well using a precise P200 pipette tip, creating a distinct gap in the cell monolayer. After the scratch, the media was replaced to eliminate debris and suspended cells, exposing the cells to the experimental conditions (control, 50, 250, and 500 pg/µL of glucagon) and control conditions. Bright-field images of each well were captured at specific timepoints (0, 10, 24, and 48 h) using the Olympus IX53 Inverted Microscope. ImageJ v.1.53 software was utilized to analyse and quantify the obtained images.

### 2.7. Western Blotting

For Western blotting, 5 × 10^5^ cells/well/2 mL were plated in a 6-well plate. After starvation, the cells were cultured for 48 h in the presence of 250 pg/mL of glucagon, with or without glucose, and control conditions. To assess the effect of the activation of MAPK signalling pathway induced by glucagon, the levels of phosphorylated extracellular signal-regulated kinase1/2 (pERK1/2) were analysed using the Western blotting technique. Cell pellets were lysed in 3-(N-morpholino)propanesulfonic acid (MOPS) buffer and stored at −20 °C. The protein concentration was determined using the Bradford method and spectrophotometric quantification at 595 nm with the Bio-Rad protein assay reagent. Samples were prepared by adding 5× loading buffer with 10% β-mercaptoethanol to each cell lysate, followed by boiling at 95–100 °C for 10 min. After centrifugation, the denatured protein samples were loaded into a 12% polyacrylamide gel and separated by electrophoresis (PAGE) using the MINI-PROTEAN Tetra Electrophoresis System. Electrophoresis was conducted in 1× TGS buffer at 140 V. Subsequently, the proteins were transferred from the gel to an Immuno-Blot PVDF membrane using the Trans-Blot Turbo Blotting system. The membrane was blocked with 5% BSA in 1× TBS containing 0.1% Tween 20 for 2 h, at RT. For protein detection, the membrane was incubated overnight at 4 °C with anti-p ERK1/2 (anti-phospho p44/42 MAPK, 9101, Cell Signalling Technology, Danvers, MA, USA, at 1:1000 concentration), in 3% BSA in 1× TBS containing 0.1% Tween 20, overnight. After washing off the unbound primary antibodies, the membrane was incubated with a secondary antibody IgG-conjugated horseradish peroxidase (HRP; anti-rabbit, 1:5000, 31460, Thermo Scientific) for 2 h, at RT. Following additional washes, the immunoreactive bands on the membrane were developed using an ECL chemiluminescent substrate and captured using a ChemiDoc XRS System with Image Lab v2.0.1 software. Total ERK1/2 was determined using the anti-p44/42 MAPK (Erk1/2; 137F5, Cell Signalling Technology, at 1:1000 concentration), in 3% BSA in 1× TBS containing 0.1% Tween 20, overnight. After washing off the unbound primary antibodies, the membrane was incubated with a secondary antibody IgG-conjugated horseradish peroxidase (HRP; anti-rabbit, 1:5000, 31460, Thermo Scientific) for 2 h, at RT. To normalize the protein levels, the membrane was subsequently probed with a mouse anti-human α-tubulin antibody (1:4000; clone B-5-1-2, T5168, Merck KGaA) followed by a secondary antibody IgG-conjugated horseradish peroxidase (HRP; anti-mouse, 1:5000, 31430, Thermo Scientific). The bands were quantified using Image J software.

### 2.8. Nuclear Magnetic Resonance (NMR) Spectroscopy

For nuclear magnetic resonance (NMR) spectroscopy, 1 × 10^5^ cells/well/500 µL were cultured in 24-well plates, and the supernatant was collected, cleared upon centrifugation (150× *g*, 5 min) and stored at −80 °C until NMR analysis. After starvation, cells were cultured in the experimental conditions for 48 h in the presence of 250 pg/mL of glucagon, without or with glucose (4.5 g/L). NMR spectroscopy was performed to define the exometabolome (metabolic profiles of conditioned culture media) under the influence of glucagon in the absence or presence of glucose in pNET cell lines and in the non-malignant α-TC1 cell line. The supernatants were collected, and centrifuged 150× *g* for 3 min, and stored at −80 °C to preserve their integrity. For sample preparation, 60 µL of a solution containing 0.022 mM trimethylsilyl propionate-d4 (TSP) and 0.04% (*v*/*v*) sodium azide, both in D_2_O, was added in a 1:1 proportion to each supernatant. TSP serves as an internal standard, providing a reference point for concentration measurements. ^1^H-NMR spectra were acquired in a 500 MHz magnetic field in the 500 UltraShieldTM Spectrometer (Bruker) using a 5 mm TCI-z (5 mm), using the noesypr1d pulse program, at 25 °C. Acquisition parameters were as follows: 128 scans, a spectral width of 11.7616 ppm, and a free induction decay (FID) of 0.244711 Hz. To process and analyse the obtained spectra, the TopSpin 4.1 software (Bruker) was used. Spectral processing techniques, such as baseline correction and referencing, were applied to enhance the quality of the spectra. Additionally, spectral assignments were performed to identify and characterise the metabolites present in the samples by resorting to spectral databases: Chenomx NMR Suite 8.11 and Human Metabolome (HMDB). Results are expressed as the net concentration of organic compounds in the conditioned culture media. The initial concentration of the same compounds in the fresh (naïve) media were considered to discuss either the augment/production or reduction/consumption upon cell culture at the different conditions.

### 2.9. Immunohistochemistry (IHC)

The expression of the GCGR and GLP-1R was analysed in 60 paired tumour and normal tissue samples. These pNET specimens were obtained from a cohort of patients (23 men and 37 women; 50% were 24–55 years old and 50% were 55–85 years old) at the Pathology Department of Hospital Curry Cabral from the Centro Hospitalar Universitário Lisboa Central (CHULC). The retrospective study evaluated surgical samples collected from 2013 and 2020 and received approval from the Ethical Committee of CHULC (REF: 1264/2022). Immunodetection was performed in 4 µm thick formalin-fixed, paraffin-embedded tissue samples, after deparaffinization and rehydration according to standard protocols [36]. The evaluation of the GCGR and GLP-1R expression in tumours and normal pancreas tissue provided information on the distribution and abundance of the GCGR and GLP-1R and on their dynamics upon malignancy. Specific primary antibodies against the GCGR and GLP-1R were used (rabbit anti-human GCGR, 1:500, ab75240, Abcam, and rabbit anti-human GLP-1R, 1:300, 1R1J8, Invitrogen), for 16 min. Pretreatment (CC1—24 min; Ventana Medical Systems, Tucson, AZ, USA) with appropriate positive and negative controls samples was conducted using the BenchMark ULTRA IHC/ISH Automatic staining platform (Ventana Medical Systems, Tucson, AZ, USA) and the OptiView DAB IHC Detection Kit with diaminobenzidine as the chromogen to detect antigen expression. Tissue sections were counterstained with Mayer’s haematoxylin before mounting. Image acquisition was performed using a Digital Microimaging Device Leica DMD108 (version 1.15 Build 704, Leica Microsystems, Wetzlar, Germany). The histopathological analysis was performed by two independent pathologists and a detection score was defined (0—absent, 1—weak, 2—moderate, and 3—strong), as represented in Figure S1.

### 2.10. Statistical Analysis

All results were analysed by GraphPad Prism 7.00 software (www.graphpad.com/), using a student’s *t*-test, one-way ANOVA, or two-way ANOVA to evaluate the statistical significance of results. The relationship between GCGR/GLP1R expression and clinicopathological features was analysed using univariate analysis (two-tailed t-test) and a binomial exact test on SPSS 24.0 software. The assays were performed with, at least, 3 replicates per condition and the differences were considered statistically significant at *p* < 0.05. A multivariate statistical analysis of ^1^H NMR data was performed on MetaboAnalyst 5.0 (assessed on 4 August 2023) using metabolite concentrations as inputs and scaled using pareto-scaling. ^1^H-NMR data were subjected to unbiased metabolic profiling using principal component analysis (PCA), and for the maximization of the metabolic differences between different groups, a partial least square analysis (PLS-DA) was performed, which can be classified as supervised multivariate statistical analysis. Heatmaps representing the univariate analysis of the extracellular levels of the different metabolites detected by NMR were created for each cell line. The parameters that were used for the analysis were the Euclidean distance measure and the Ward cluster algorithm, using MetaboAnalyst 5.0 software.

## 3. Results

### 3.1. Glucagon-Induced GCGR Signalling Promotes pERK1/2 Activation in pNET Cells, Mainly Under Glucose Deprivation

**Figure 1:**
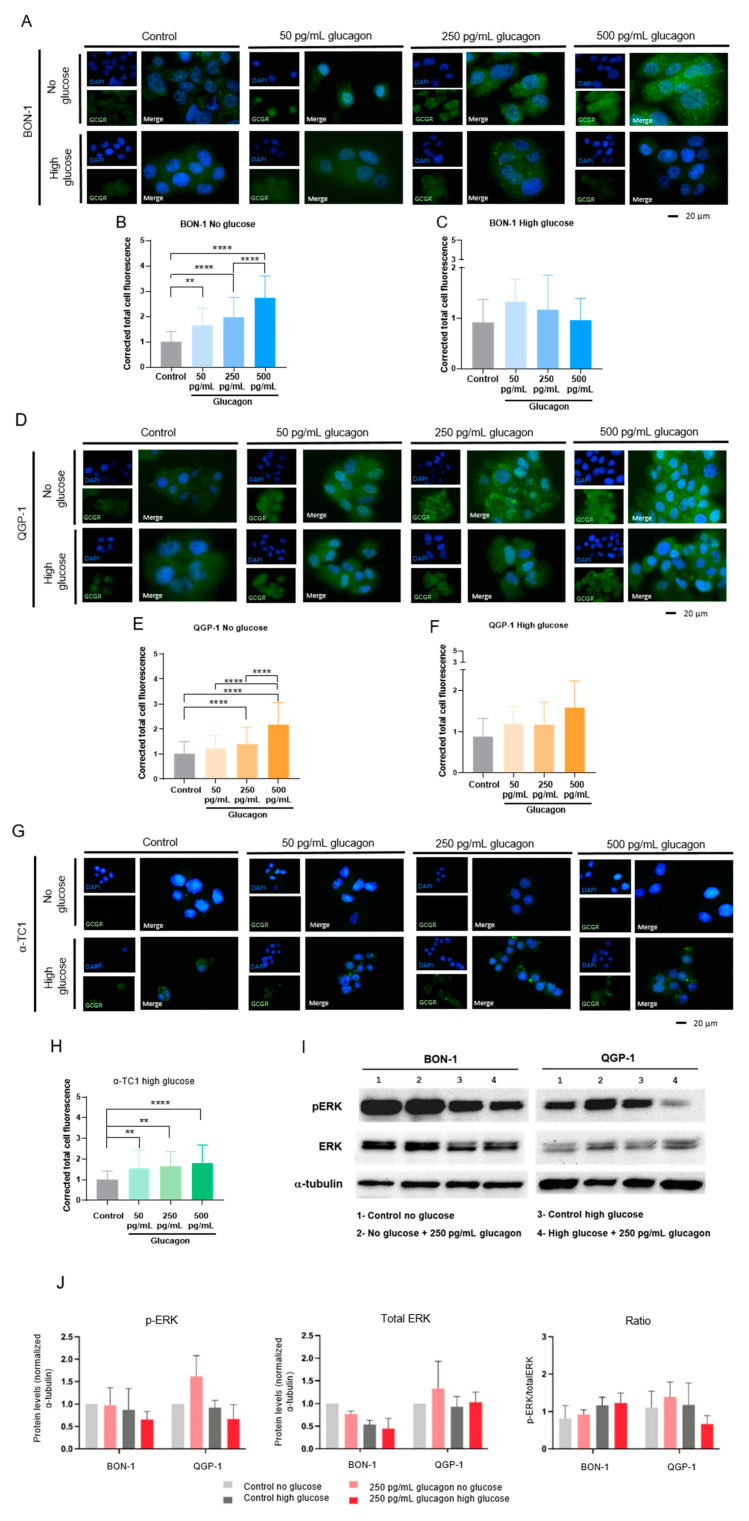
Glucagon-induced glucagon receptor (GCGR) signalling promotes mitogen-activated protein kinase/ extracellular signal-regulated kinase (MAPK/ERK) signalling cascade activation in pNET malignant cells under glucose deprivation and in non-malignant cells under high glucose levels. (**A**) Immunofluorescence staining of GCGR in BON-1 cells cultured in DMEM-F12 without glucose or with high glucose levels (25 mM) for 24 h and treated with different glucagon concentrations, simulating normal physiological levels (50 pg/mL) and hyperglucagonemia-compatible concentrations (250 and 500 pg/mL). In control conditions, cells were cultured without glucagon. A primary antibody against GCGR (1:500; ab75240, Abcam) was used followed by secondary goat anti-rabbit Alexa Fluor^®^ 488 (green) conjugated antibody (1:1000, A-11078, Invitrogen—Thermo Fisher Scientific). Nuclei were stained blue with DAPI. Images were captured at 400× magnification using an Axio Imager.Z1 microscope (Zeiss). (**B**) Quantification of GCGR expression in BON-1 (**C**,**D**) and QGP-1 (**E**,**F**) cells cultured without glucose and in high-glucose conditions, respectively. Fluorescence quantification was performed by corrected total cell fluorescence, Image J. (**G**) Immunofluorescence staining of glucagon receptor in immortalized pancreatic α-cells (α-TC1) in DMEM cultured in the same experimental conditions as in (**A**). In control conditions, cells were cultured without glucagon. (**H**) Quantification of GCGR expression in α-TC1 cells cultured in high-glucose conditions, measured by corrected total cell fluorescence. (**I**) Western blotting for pERK1/2 and total ERK1/2 detection on BON-1, QGP-1, and αTC-1 cell lines. The values are normalized for the internal control, α-tubulin. (**J**) Western blotting quantification, using Image J, of BON-1 and QGP-1, respectively. Lane 1—control without glucose and without glucagon; lane 2—medium without glucose and 250 pg/mL glucagon; lane 3—control with high glucose levels and without glucagon; and lane 4—medium-glucose levels and 250 pg/mL glucagon. All data are presented as mean ± standard deviation (SD). Statistical analysis was performed using one-way ANOVA. ** *p* < 0.001, and **** *p* < 0.00001 indicate a significant difference compared to control.

The expression of the GCGR in pNET cell lines and the effects of glucagon on pNET and non-cancer cells were investigated. Interestingly, both BON-1 and QGP-1 cell lines displayed a significant increase in GCGR expression under glucose-deprived conditions, with the most prominent effect observed at 500 pg/mL of glucagon (Figure 1A,B,D,E). In high-glucose conditions, BON-1 cells did not show significant differences in GCGR expression independent of glucagon exposure (Figure 1A,C). In contrast, QGP-1 cells exhibited a trend towards increased GCGR expression with higher glucagon concentrations under high-glucose conditions (Figure 1D,F). In the non-malignant pancreatic α-TC1 cells, no significant differences in GCGR expression were observed at different glucagon concentrations in the absence of glucose (Figure 1G). However, a significant increase in GCGR expression was observed in high-glucose conditions, particularly at a concentration of 500 pg/mL of glucagon (Figure 1G,H).

The activation of the GCGR signalling pathway following exposure to glucagon was investigated; hence, the levels of pERK1/2 were assessed by Western blotting analysis (Figure 1I,J). In glucose-deprived conditions, no alteration was observed in pERK1/2 levels in BON-1 cells, but an increase in pERK1/2 levels upon glucagon exposure was observed in QGP-1 cells, compared with the control condition without glucagon. In the presence of glucose, both cell lines showed a decrease in pERK1/2 levels. These findings highlight that glucagon may confer adaptive advantages to pNET cells under conditions of glucose scarcity. The same trend was found in QGP-1 when analysing the pERK1/2 versus total ERK1/2 (Figure 1J).

### 3.2. Glucagon Impacts pNET Cell and α-TC1 Cell Features Differently

**Figure 2:**
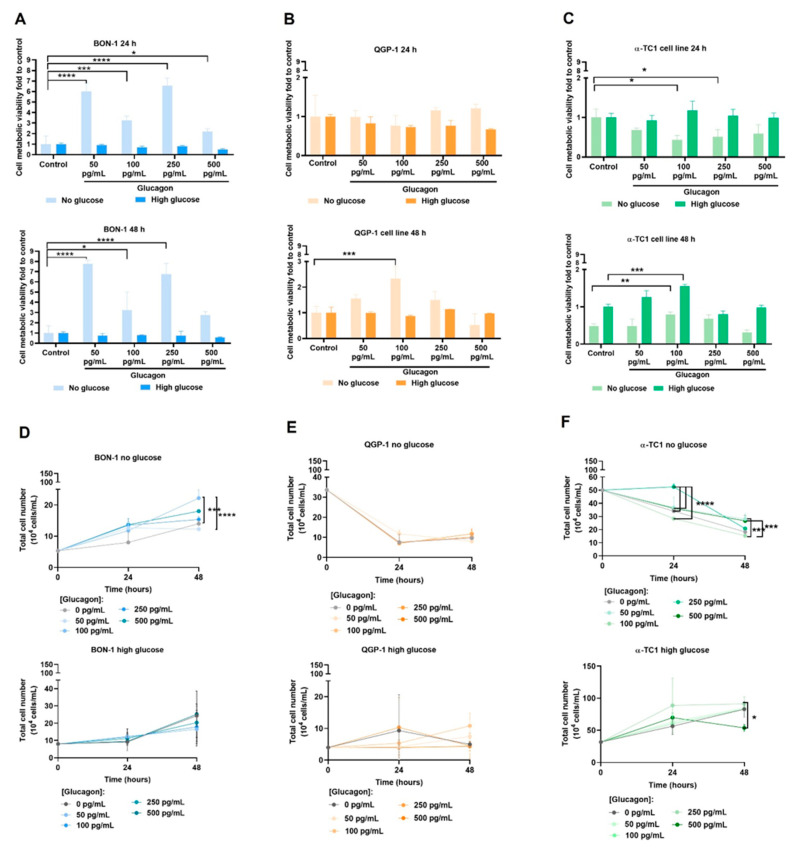

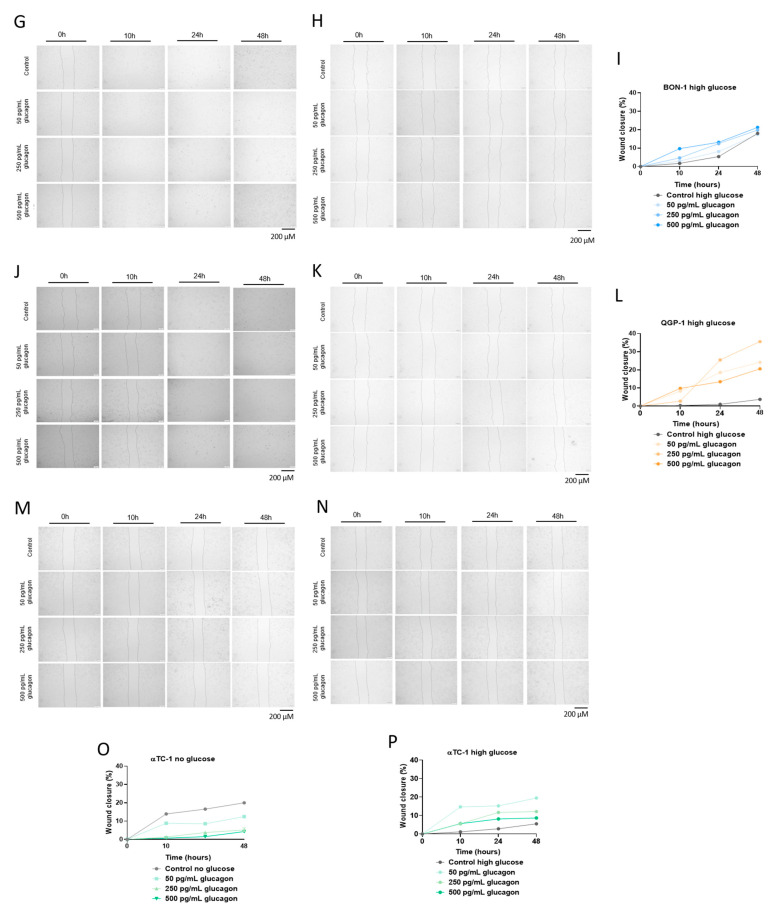
Hyperglucagonemia-compatible concentration promotes pNET BON-1 cell viability under glucose deprivation, while glucagon drives migration in pNET cells and in non-malignant pancreatic α-TC1 cells under high-glucose conditions. The metabolic viability assay performed on (**A**) BON-1 cells, (**B**) QGP-1 cells, and (**C**) α-TC1 cells cultured in DMEMF12 without or with high glucose levels (25 mM) for 24 and 48 h, and treated with varying glucagon concentrations, simulating normal physiological levels (50 and 100 pg/mL) and hyperglucagonemia-compatible concentrations (250 and 500 pg/mL) using the Cell Counting Kit-8 (CCK-8) assay. In control conditions, cells were cultured without glucagon. Absorbance was measured at 450 nm using a microplate reader. Proliferation assay performed on (**D**) BON-1 cells, (**E**) QGP-1 cells, and (**F**) α-TC1 cells cultured in DMEMF12 without or with high glucose levels (25 mM) for 24 and 48 h, and treated with varying glucagon concentrations (50, 100, 250, and 500 pg/mL), using a cell counter chamber. Representative microscope images (400 magnification, scale: 200 µM) of the wound closure evolution throughout 48 h of (**G**) BON-1cells without glucose; (**H**) BON-1 cells under high-glucose conditions; (**J**) QGP-1 cells without glucose; (**K**) QGP-1 cells under high-glucose conditions; (**M**) α-TC1 cells without glucose; and (**N**) α-TC1 cells under high-glucose conditions. Cells were exposed to varying glucagon concentrations (50, 100, 250, and 500 pg/mL) in the absence of glucose. Quantitative analysis of the wound closure of the (**I**) BON-1 cells and (**L**) QGP-1 cells at 50, 100, 250, and 500 pg/mL of glucagon, under high-glucose conditions. Quantitative analysis of the wound closure of the α-TC1 cell line (**O**) without glucose and (**P**) under high-glucose conditions. All data are presented as mean ± standard deviation (SD). Statistical analysis was performed using one-way ANOVA. * *p* < 0.05, ** *p* < 0.001, *** *p* < 0.0001, and **** *p* < 0.00001 indicate a significant difference compared to control.

The impact of glucagon and glucose was investigated on various cellular features, including metabolic cell viability, cell proliferation, and cell migration, in pNET cell lines and α-TC1 cells. By using the control condition (no glucose and no glucagon) as a reference, it was observed that under glucose-deprived conditions, glucagon confers increased BON-1 cell viability, with the highest viability observed at concentrations of 50 pg/mL and at 250 pg/mL, after 24 and 48 h of treatment (Figure 2A). No differences were observed in the cell viability of BON-1 cells cultured in high-glucose conditions without or with glucagon (Figure 2A). QGP-1 cells in glucose-deprived conditions did not show any significant differences regarding the presence of glucagon at 24 h of culture, but at 48 h, a significant increase in viability was observed in cells exposed to 100 pg/mL of glucagon (Figure 2B). No differences were observed in QGP-1 cells cultured in high-glucose conditions and exposed to glucagon, after 24 h and 48 h (Figure 2B). In the α-TC1 cell line, the 24 h assay demonstrated a decrease in cell viability with glucagon, with significant differences at 100 pg/mL and 250 pg/mL of glucagon under high-glucose conditions (Figure 2C). No differences were observed in the absence of glucose (Figure 2C). After 48 h, 100 pg/mL of glucagon significantly increased cell viability under both glucose-deprived and high-glucose conditions (Figure 2C).

Regarding cell proliferation, BON-1 cells exhibited a significant increase in proliferation at concentrations of 100 pg/mL and 500 pg/mL of glucagon under glucose-deprived conditions, while no significant changes were observed in high-glucose conditions (Figure 2D). In QGP-1 cells, no significant differences between culture conditions were observed independent of glucose presence or absence. However, glucose-deprivation induced a decreased proliferation rate over time across all conditions, independent of glucagon concentration, at either 24 or 48 h (Figure 2E). In α-TC1 cells, a trend to a time-dependent increase in proliferation was observed for all concentrations of glucagon in the presence of glucose, being significant at 250 pg/mL of glucagon. In the absence of glucose, a significant decrease in cell proliferation was observed over time (Figure 2F).

The in vitro wound healing assay was conducted to evaluate the effect of glucagon on the two-dimensional migration rate of pNET cells and α-TC1 cells. In no-glucose conditions, no significant differences were detected in pNET cell lines (Figure 2G–M). In high-glucose conditions, BON-1 cells tended to migrate more with 500 pg/mL of glucagon, but no significant differences were observed (Figure 2I). In high-glucose conditions, QGP-1 cells migrated more in the presence of glucagon compared to control (Figure 2L). Under glucose deprivation, after 24 h, a similar effect was observed. In α-TC1 cells, under glucose-deprived conditions, the control group displayed the highest degree of wound closure (Figure 2O). However, in high-glucose conditions and under glucagon exposure, cells migrated more than in the control conditions (Figure 2P).

These findings suggest that the non-malignant pancreatic (α-TC1) cell line is highly dependent on glucose to sustain survival and proliferation, while the pNET (BON-1 and QGP-1) cell lines, as previously observed, are probably metabolically distinct and respond differently to glucagon concentrations.

### 3.3. Glucagon Impacts pNET Cell and α-TC1 Cell Metabolic Remodelling Differently, Depending on Glucose Availability

**Figure 3:**
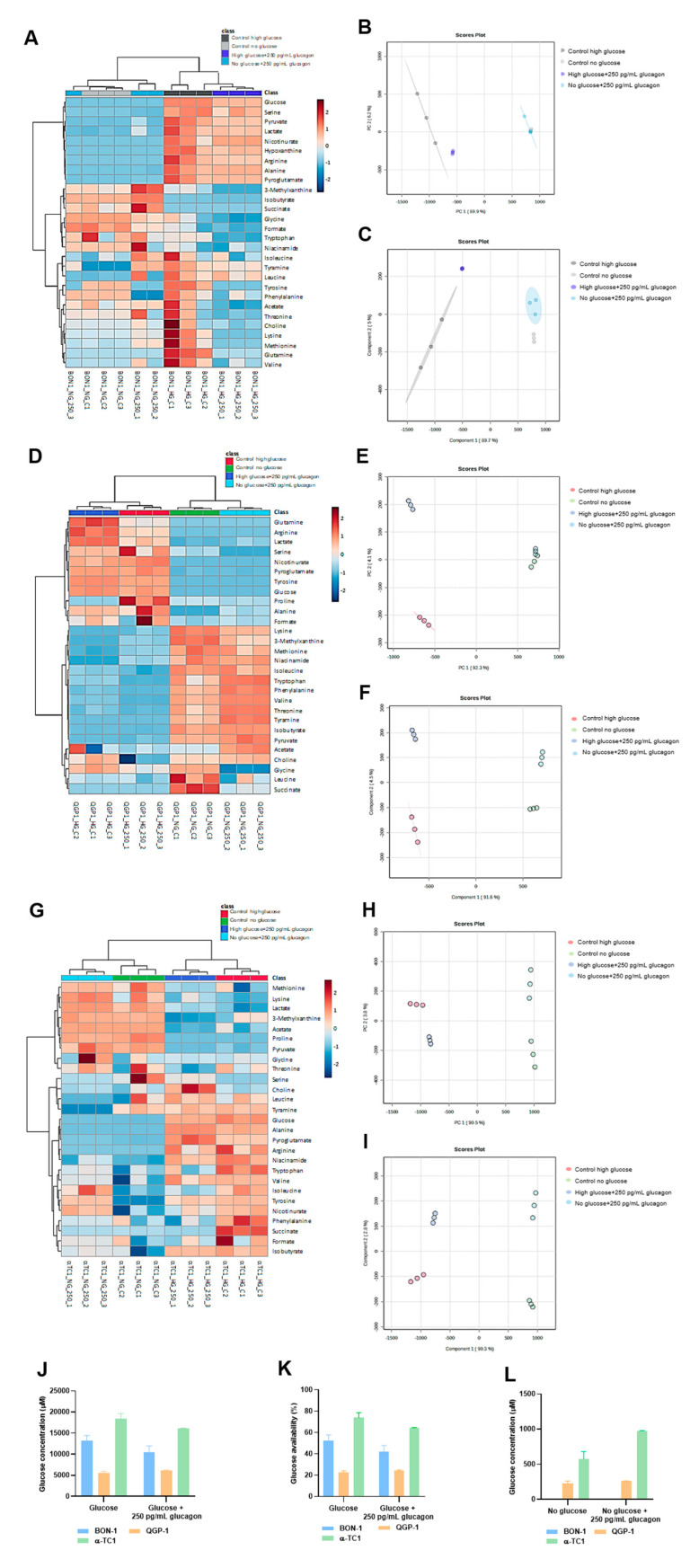
Glucagon impacts BON-1, QGP-1, and α-TC1 cells differently; pancreatic neuroendocrine tumour (pNET) cell lines consume more glucose than non-cancer α-TC1 cells, which are efficient producers of glucose stimulated by glucagon. (**A**,**D**,**G**) Heatmaps showing glucose and glucagon pathway clustering for BON-1, QGP-1, and α-TC1, respectively. (**B**,**E**,**H**) Principal component analysis (PCA) to infer the influence of glucose and glucagon on clustering patterns in BON-1, QGP-1, and α-TC1, respectively. (**C**,**F**,**I**) Partial least-squares discriminant analysis (PLS-DA) to infer glucose and glucagon influence on clustering patterns, in BON-1, QGP-1, and α-TC1, respectively. (**J**) BON-1, QGP-1, and α-TC1 cell lines’ glucose consumption. (**K**) BON-1, QGP-1, and αTC1 cell lines’ percentage of glucose consumption. (**L**) BON-1, QGP-1, and α-TC1 glucose production in glucose-deprived medium. Statistical analysis was performed using one-way ANOVA.

The heatmaps for metabolic profiles, PCA analysis, and PLS-DA analysis show visible metabolic shifts in BON-1 (Figure 3A–C), QGP-1 (Figure 3D–F) and α-TC1 (Figure 3G–I) cell lines, indicating glucagon modulation according to glucose availability. The PLS-DA were performed using the metabolite concentrations determined by ^1^H-NMR, with an R^2^ = 0.93933 and Q^2^ = 0.81207, R^2^ = 0.972881 and Q^2^ = 0.93257, and R^2^ = 0.99034 and Q^2^ = 0.88941, for BON-1, QGP-1, and α-TC1, respectively. Permutation tests were performed and validated the analysis (Figure S2).

The exometabolome of BON-1 and QGP-1 was explored, and 30 metabolites were identified and quantified. In high-glucose conditions, arginine and glutamine were produced, and it was dependent on glucagon in BON-1 and independent of glucagon in QGP-1 (Figure 3A,D and Figure S3A,B). Also, in high-glucose conditions, alanine, pyroglutamate, and serine levels were significantly increased compared to no-glucose conditions, in both cell lines and independently of glucagon exposure (Figure 3A,D and Figure S3A,B). Regarding other amino acids, BON-1 did not present more alterations between culture conditions (Figure 3A and Figure S3A). In QGP-1, a significant increase in glycine and proline levels was observed, and there was a decrease in isoleucine, lysine, methionine, phenylalanine, threonine, tryptophan, and valine levels, independently of glucagon (Figure 3D and Figure S3B). Notably, BON-1 is more responsive to glucagon than QGP-1 in respect to amino acids metabolism when glucose is available.

In no-glucose conditions, the levels of organic acids presented alterations, namely isobutyrate was increased in BON-1 and isobutyrate and pyruvate were increased in QGP-1, comparing to cells cultured in high-glucose conditions (Figure 3A,D and Figure S3D,E). Lactate levels were increased in BON-1 and QGP-1 cells exposed to high-glucose conditions, and in QGP-1 cells, glucagon contributed to this increment (Figure 3D and Figure S3E). Hypoxanthine was only detected in QGP-1 cells exposed to high glucose levels, independent of glucagon (Figure S3E). Methylxanthine levels were decreased in high-glucose conditions, in BON-1 and QGP-1 cell lines (Figure S3G–I). In BON-1, tyramine was not detected in no-glucose control conditions, but its levels raised upon glucagon exposure (Figure S3G). In QGP-1, tyramine was not detected in high-glucose conditions independently of glucagon (Figure S3H). In glucose scarcity, both BON-1 and QGP-1 present metabolic adjustments regarding the organic acids, and glucagon regulates the increased production of lactate in QGP-1. Furthermore, glucagon regulates tyramine production in BON-1, which is related to its function as a serotonin producer.

In α-TC1 cells, 28 metabolites were identified and quantified using NMR analysis. When comparing cells exposed to high-glucose conditions to those cultured without glucose, we observed increased levels of alanine, arginine, and pyroglutamate, and decreased levels of lysine (Figure 3G and Figure S3C). Importantly, only the increase in pyroglutamate levels was specifically due to glucagon exposure under high-glucose conditions (Figure 3G and Figure S3C). Regarding organic acids in α-TC1 cells, in high-glucose conditions, isobutyrate and succinate levels were increased, while acetate, lactate, and pyruvate levels were decreased compared to cells in no-glucose conditions (Figure 3G and Figure S3F). Upon glucagon exposure, in high-glucose conditions, succinate was not detected (Figure S3F). Methylxanthine levels were decreased in high-glucose conditions, in α-TC1 cells (Figure S3G–I). Moreover, tyramine was not detected in these cells upon glucagon exposure in no-glucose conditions (Figure S3I).

### 3.4. pNET Cells Are Heavy Consumers of Glucose, with QGP-1 Cells Being Able to Produce Glucose, While in α-TC1 Cells, Glucagon Regulates the Consumption and Production of Glucose

In all cell lines, the consumption of glucose was assessed by calculating the difference between the ^1^H-NMR-quantified glucose levels in cells cultured under high-glucose conditions, in the beginning and in the end of the assay. QGP-1 cells demonstrated the highest efficiency in glucose consumption, utilizing approximately 80% of the available glucose, with no discernible impact from glucagon (Figure 3J,K). BON-1 and α-TC1 cells presented 50 and 70% of the initial glucose, corresponding to a consumption of 50 and 30%, respectively (Figure 3J,K). In α-TC1 cells, glucagon increased glucose consumption by approximately 10% (Figure 3J,K). The most interesting results were regarding glucose production, assessed in no-glucose culture conditions, by calculating the difference between the ^1^H-NMR-quantified glucose levels in cells cultured under high-glucose conditions, in the beginning and in the end of the assay. Glucose was not detected in BON-1 cells, but QGP-1 and α-TC1 were able to produce glucose (Figure 3L). α-TC1 cells exhibited the highest efficiency in glucose production, which was further stimulated by glucagon to about 2-fold (Figure 3L).

### 3.5. Gluconeogenesis Occurs in pNET Cells Independently of Glucose Availability and upon Hyperglucagonaemia-Compatible Concentrations

In this section, we outline the main alterations in gene expression observed in pNET cell lines and in α-TC1 cells under different conditions, including variations in glucose availability and glucagon stimulation.

**Figure 4:**
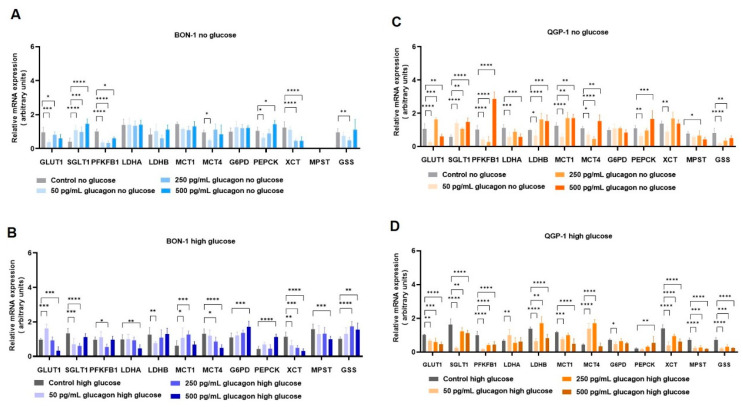
Hyperglucagonemia-compatible concentration activated gluconeogenesis in BON-1 and QGP-1 cells independently of glucose availability. (**A**,**C**) Relative gene expression of relevant genes and transporters in a pancreatic neuroendocrine tumour (pNET) context in glucose-deprived conditions, in BON-1 and QGP-1 cell lines. (**B**,**D**) Relative gene expression of relevant genes and transporters in a pNET context in high-glucose conditions, in BON-1 and QGP-1. Cells were cultured in control and 50, 250, and 500 pg/mL glucagon conditions. The HPRT gene was used as an endogenous control. Expression levels were normalized to the control condition. Statistical analysis was performed using two-way ANOVA. * *p* < 0.05, ** *p* < 0.01, *** *p* < 0.001, and **** *p* < 0.0001 indicate a significant difference between each mean of each group.

In BON-1 cells cultured in no-glucose conditions (Figure 4A), an attempt to increase the uptake of glucose was observed by an increased expression of the *SGLT1/SLC5A1* gene. The decreased rate of glycolysis is demonstrated by the decreased expression of the *PFKFB1* gene, and no alterations were observed due to glucagon in the expression of *LDHA* and *LDHB* genes, which are responsible for the interconversion of lactate ↔ pyruvate [37]. No alteration was observed in *MCT1/SLC16A1* gene expression, which is more linked to lactate import, and decreased levels of *MCT4/SLC16A4* were observed, being a gene associated with lactate export. The expression of the *G6PD* gene, belonging to PPP, was not altered by glucagon, and *PEPCK* from gluconeogenesis was increased by a hyperglucagonemia-compatible concentration (500 pg/mL). BON-1 cultured in high-glucose conditions (Figure 4B) demonstrated increased levels of the *GLUT1/SLC2A1* gene upon exposure to 50 pg/mL of glucagon. Additionally, glucagon induced a decreased expression of *LDHA* and *LDHB* of 50 and 500 pg/mL, respectively. These results suggest that a hyperglucagonemia-compatible concentration could favour the consumption of lactate, while physiological levels of glucagon could favour the production of lactate. This interpretation is supported by the roles of LDHA in lactate production and LDHB in the conversion of lactate to pyruvate [38]. Accordingly, *MCT1/SLC16A1* expression increased and *MCT4/SLC16A4* expression decreased with glucagon (50 and 500 pg/mL), since MCT1 is more related to the import, and MCT4 to the export, of lactate [39]. Regarding PPP and gluconeogenesis, glucagon tended to increase the expression of limiting genes, respectively, the *G6PD* and *PEPCK* genes, being significant at 500 pg/mL.

In QGP-1 cells cultured in no-glucose conditions (Figure 4C), glucagon increased *GLUT1/SLC2A1* and *SGLT1/SLC5A1* expression. Moreover, QGP-1 cells presented a decreased expression of glycolytic gene *PFKFB1* at 50 and 250 pg/mL of glucagon, with an increase in PFKFB1 expression at 500 pg/mL of glucagon. *LDHA* and *MCT4/SLC16A4* levels were decreased by glucagon (50 and 250 pg/mL), while *LDHB* and *MCT1/SLC16A1* levels were increased at the same concentrations, suggesting the activation of lactate consumption. The PPP gene *G6PD* presented no alterations, while the gluconeogenesis gene *PEPCK* was increased at concentrations compatible with hyperglucagonemia. QGP-1, in high-glucose conditions (Figure 4D), showed decreased levels of *GLUT1/SLC2A1*, *SGLT1/SLC5A1,* and *PFKFB1* gene levels, suggesting this cell line may be less dependent on glucose than the BON-1 cell line. Moreover, *LDHA* was increased by physiological levels of glucagon and *LDHB* was increased in hyperglucagonemia-compatible concentrations, which fits with the decreased levels of *G6PD* at physiological levels of glucagon, since decreased glycolysis produces less glucose-6-phosphate to supply PPP. *MCT1/SLC16A1* was decreased and *MCT4/SLC16A4* was increased by glucagon, reinforcing the low rate of glycolysis, indicating that lactate import is less relevant in QGP-1. Additionally, the expression of the *PEPCK* gene from gluconeogenesis was increased by hyperglucagonemia-compatible concentrations.

Cysteine and oxidative stress-related metabolism was also assessed by the expression of genes encoding the *xCT/SLC7A11* transporter; MST, an enzyme from cysteine catabolism; and GSS, responsible for glutathione synthesis. In BON-1 and QGP-1, *xCT/SLC7A11* expression was decreased by glucagon independent of glucose (Figure 4A–D). The same was verified for *MPST* expression, in QGP-1, but in BON-1, *MPST* was not detected in no-glucose conditions (Figure 4A–D). *GSS* tended to decrease in BON-1 and QGP-1 cultured in no-glucose conditions upon glucagon exposure, while in high-glucose conditions, *GSS* levels increased in BON-1 and decreased in QGP-1 with glucagon (Figure 4A–D).

### 3.6. GCGR and GLP-1R Expression Is Lower in pNETs Compared to Normal Pancreatic Tissue, Highlighting a Prognostic Role of These Receptors

We aimed to investigate the correlation between GCGR and GLP-1R expression and various clinical parameters in pNETs. Immunohistochemistry was performed on 60 cases, composed of paired tumour and normal tissue samples, and the clinical/biological parameters were assessed and presented in Table 2 and Figure S1. Out of the 60 cases, 91.7% (55/60) were GCGR-positive and 30% (18/60) were GLP1R-positive. There is a significant difference in the expression levels of GCGR between males and females (*p* = 0.023) and the same was observed for GLP1R (*p* = 0.014). Moreover, GCGR significantly correlated with the intensity staining score (*p* = 0.023), in which in the positive samples for GCGR, 31 samples (86.1%) were scored with 0–1 and 24 (100%) were scored with 2/3. The other variables were not significantly associated with GCGR or GLP-1R.

**Figure 5:**
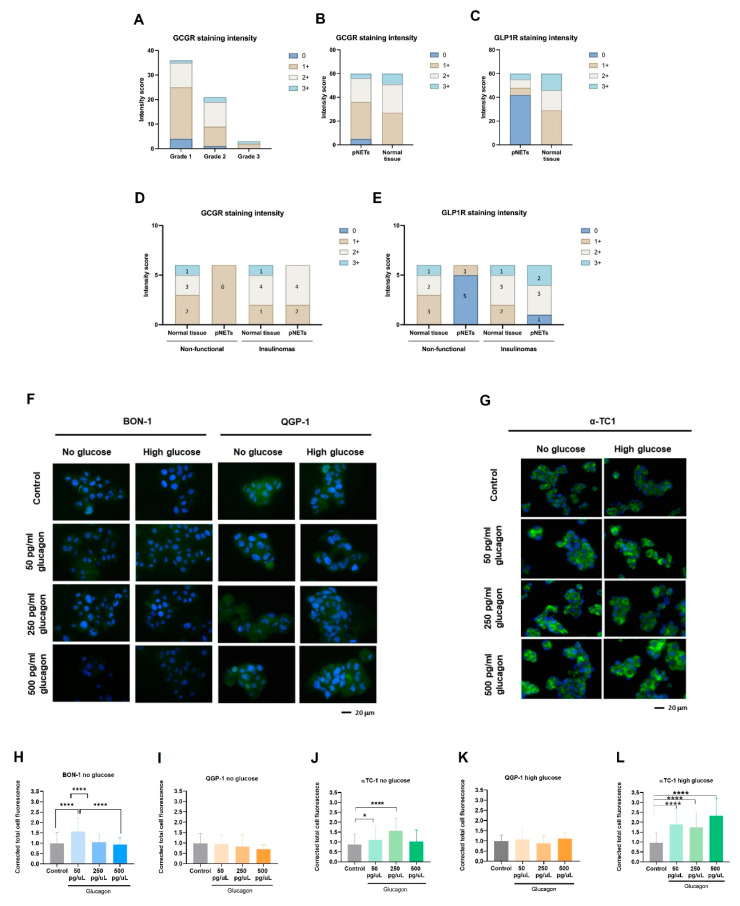
Glucagon receptor (GCGR) staining intensity decreases with tumour grading and glucagon-like peptide-1 receptor (GLP-1R) expression is higher in pancreatic neuroendocrine tumours (pNETs) compared to normal pancreas tissue, highlighting a putative prognostic role of these receptors. (**A**) Staining intensity of GCGR in different pNET grades. Immuno-staining intensity scores for (**B**) GCGR and (**C**) GLP-1R in different pNET grades, obtained by immunohistochemistry. Comparison between non-functional pNETs and insulinomas of (**D**) GCGR and (**E**) GLP-1R staining intensity. Immunofluorescence for GLP-1 receptor (GLP-1R; green) in (**F**) BON-1 and QGP-1 cells, and (**G**) α-TC1 cells cultured without glucose or in high-glucose conditions for 24 h and treated with different glucagon concentrations, simulating normal physiological levels (50 pg/mL) and hyperglucagonemia-compatible concentrations (250 and 500 pg/mL). In control conditions, cells were cultured without glucagon. (Nuclei were stained blue with DAPI. Images were captured at 400× magnification using an Axio Imager.Z1 microscope (Zeiss). Quantification of GLP-1R expression in (**H**) BON-1, (**I**) QGP-1, and (**J**) α-TC1 cells cultured in glucose scarcity conditions and (**K**) QGP-1 and (**L**) α-TC1 cells cultured in high-glucose conditions, respectively. Fluorescence quantification was performed by corrected total cell fluorescence, Image J. All data are presented as mean ± standard deviation (SD). Statistical analysis was performed using one-way ANOVA and univariate analysis (two-tailed *t*-test) on SPSS software v.28.0; * *p* < 0.05, and **** *p* < 0.0001 indicate a significant difference compared to control.

When evaluating the tumour grade, we observed that most grade 1 cases exhibited weak to moderate levels of GCGR, while grade 2 cases showed slightly more intense staining (Figure 5A). However, grade 3 cases showed greater variability, with both weak and strong GCGR staining intensities observed. Future studies involving a larger cohort may provide more reliable conclusions on the relationship between GCGR expression and pNET grade.

In addition to GCGR expression, we also assessed the expression of GLP-1R, the receptor for GLP1, which inhibits glucagon secretion. Immunohistochemistry analysis revealed a significantly different pattern of GLP-1R expression between pNETs and normal tissue (Table 3, Figure 5C and Figure S1). pNETs express lower levels of GLP-1R, compared to normal tissue (Figure 5C). Indeed, the expression of GPL-1R was significantly associated with a higher intensity score in normal pancreatic tissue (*p* < 0.001). Among these cases, few pNETs were functionally characterized, with six identified as non-functional and six as insulinomas. Regarding the expression of the GCGR and GLP-1R in these cases, a significant decrease in intensity staining for the GCGR and GLP-1R was observed in non-functional pNETs compared to normal pancreas tissue (Figure 5D,E), while in insulinomas, pNETs showed high-intensity staining for GLP-1R similar to normal pancreas tissue (Figure 5E). Therefore, 83.3% of insulinomas were positive for both the GCGR and GLP-1R. Nevertheless, by considering the expression of the two receptors in all pNET cases, we verified that 91.6% and 30% were, respectively, positive for the GCGR and GLP-1R (Table 4), indicating a significant association of GCGR expression with malignancy. Interestingly, among the 70% of GLP-1R-negative cases 92,8% were positive for GCGR, supporting the negative correlation between GLP-1R and GCGR (Table 4).

These findings suggest a downregulation of GLP-1R upon malignancy. To further explore if a glucagon-enriched microenvironment would interfere with GLP-1R expression, we conducted 24 h in vitro studies using the same set-up used. Interestingly, BON-1 and QGP-1 cells expressed significantly lower levels of GLP-1R compared to α-TC1 cells, agreeing with the immunohistochemistry results in pNET cases (Figure 5F,G). In BON-1 cells, in the absence of glucose, there was a significant increase in GLP-1R expression under physiological glucagon conditions (Figure 5H). However, in QGP-1 cells, no significant differences were observed between the conditions (Figure 5I,J). Notably, in α-TC1 cells, in the absence of glucose, there was a significant increase in GLP-1R expression under a hyperglucagonemia-compatible concentration (250 pg/mL glucagon), compared to the control (Figure 5K). Moreover, under high-glucose conditions, GLP-1R expression was significantly increased in all conditions, particularly in a hyperglucagonemia-compatible concentration (500 pg/mL glucagon) (Figure 5L). These findings highlight that the complex interplay between glucose, glucagon, and GLP-1 signalling pathways occurs differently in malignant and non-malignant cells.

## 4. Discussion

Pancreatic neuroendocrine tumours (pNETs) are challenging to diagnose because most cases are asymptomatic. Therefore, it is crucial to deepen our understanding of the biology of this disease to identify novel biomarkers and therapeutic targets. Given glucagon’s role as a metabolic regulator, it is essential to investigate how systemic metabolism and dietary factors influence tumour progression. Consequently, the role of glucagon in cancer cell survival and tumour progression is underexplored, despite its potential as a regulator of cellular metabolism in predominant organ systems. Our findings contribute to filling this gap, demonstrating that glucagon’s interactions with both the GCGR and GLP-1R add complexity to its role in cancer biology, potentially driving adaptive mechanisms in pNET cells [40,41,42,43]. This study used pNETs as a cancer model to explore how glucagon and glucose affect cancer cell metabolism and malignancy. This approach is irrespective of whether a pNET is a glucagonoma, as the majority of pancreatic pNETs develop from hormone-producing cells within the islet of Langerhans, exposing them to a glucagon-rich environment. Therefore, glucagon concentrations reflective of both physiological levels and hyperglucagonemia-related glucagonoma syndrome were tested, providing insights across a wide range of glucagon exposure. Our results confirm GCGR expression in pNET cell lines (BON-1 and QGP-1), with significant upregulation observed under glucose deprivation, especially in the presence of hyperglucagonemia-compatible concentrations (Figure 1A–E). This suggests a potential compensatory response, whereby GCGR signalling may promote metabolic adaptations under nutrient-limited conditions, helping pNET cells meet their energy needs. Therefore, pNET cells can take advantage of the GCGR function in the regulation of glucose homeostasis and lipid metabolism, as it is described in hepatocytes, adipocytes, skeletal muscle cells, and pancreatic islet β-cells [44,45]. We further investigated GCGR expression in non-malignant pancreatic α-TC1 cells, the primary producers of glucagon, and observed significant expression under both high-glucose conditions and the three concentrations of glucagon (Figure 1G,H). This observation suggests a potential feedback loop, wherein increased GCGR expression may regulate glucagon secretion or influence the overall endocrine balance within the pancreatic islet, as demonstrated by Yagi et al. [14]. These findings collectively highlight the dynamic interplay between glucagon and glucose in modulating cellular responses, reinforcing glucagon’s role as a survival signal under metabolic stress.

The MAPK pathway is prominently stimulated by glucagon, leading to the activation of ERK1/2 [15,46], indicating a potential mechanism by which GCGR signalling impacts cellular responses within the pancreatic islet [47]. It was observed that upon glucagon stimulation, QGP-1 cells presented increased pERK1/2 levels under glucose deprivation with no alterations in the pERK1/2 / total ERK1/2 ratio (Figure 1I,J). However, when glucose was available, the levels of pERK1/2 tended to reduce upon glucagon exposure in both cell lines, suggesting a regulatory role of glucose in glucagon signalling through the ERK pathway (Figure 1I,J), which must be explored. This interplay suggests that in nutrient-scarce environments, glucagon signalling could activate pathways that aid survival by enhancing adaptive metabolism [8]. On the contrary, when glucose is available, cells need less extracellular survival cues, given their existing energy substrate. This is supported by the decreased pERK1/2 response to glucagon in the presence of glucose [7]. Glucagon plays a role in the metabolic remodelling underlying pNET cell adaptation to stressful conditions.

The differential effects of glucagon on cell viability, proliferation, and migration between BON-1 and QGP-1 cells provided further evidence of its role in pNET biology. Upon glucose deprivation, glucagon significantly increased BON-1 cell metabolic viability at all concentrations (Figure 2A). In the same conditions, BON-1 cell proliferation was increased with glucagon (Figure 2D), underscoring glucagon’s role as a potential mitogenesis stimulator [14]. Overall, these findings suggest a glucagon-triggered pro-survival and adaptive mechanism to rescue viability and sustain cell proliferation under glucose deprivation, aligning with the GCGR expression and pERK1/2 levels observed in these conditions, as well as the fact that this cell line is unable to produce glucose in order to compensate its scarcity.

Regarding QGP-1 cells, no differences were observed due to glucagon exposure, except a significant rescue of cell viability, after 48 h at 100 pg/mL of glucagon (Figure 2B). These viability findings align with the overall reduced proliferation in the absence of glucose but not in glucose-rich conditions over time (Figure 2E). Our results indicate that QGP-1 cells possess mechanisms to overcome glucose scarcity, supported in part by their ability to produce glucose (Figure 3L). This variability between cell lines indicates distinct mechanisms by which BON-1 and QGP-1 take advantage of glucagon stimulus.

The different responses of pNET cell lines to glucagon reflect the imbalance between the need for glucose and the ability to adapt to the lack of glucose. This is managed differently by these two cell lines, which showed metabolic remodelling and different abilities for consuming and producing glucose.

In terms of cell migration, under glucose scarcity, both cell lines were unable to close the wound mainly due to decreased adherent capacity. Despite QGP-1 cells also detaching under glucose-deprived conditions, this occurred later compared to BON-1 cells. Overall, QGP-1 cells exhibited higher migratory capacity than BON-1 cells, especially at hyperglucagonemia-compatible concentrations (Figure 2J–L). In glucose-rich conditions, BON-1 cells showed increased wound closure at hyperglucagonemia-compatible concentrations (Figure 2G–I). Given that BON-1 cells exhibit reduced GCGR levels in high-glucose conditions, these results imply that in the presence of scarce receptors, high glucagon concentrations more efficiently trigger a cellular migratory response. While the low migratory capacity of BON-1 and QGP-1 cell lines has been previously described [26], the role of glucagon in pNET migration has not been addressed until now.

α-TC1 cells showed a direct correlation between viability and glucagon levels in high-glucose conditions (Figure 2C). This emphasizes glucagon’s physiological role in promoting the survival and function of α-cells, especially when glucose is abundant [15,48]. However, the decreased viability in the absence of glucose at 24 h might indicate that these cells are dependent on glucose as a metabolic source (Figure 2C). The significant increase in cell viability at 48 h, even under glucose deprivation, suggests an adaptive pro-survival response (Figure 2C) and it can also be related to the adjustments these cells must undergo to produce glucose, as was observed (Figure 3L). This is in line with the proliferation increase in no-glucose conditions under 500 pg/mL of glucagon (Figure 2F). In high-glucose conditions, the proliferation of α-TC1 cells increases across most glucagon concentrations, suggesting a synergetic effect of glucose and glucagon in supporting α-cell proliferation (Figure 2F). However, a decrease in proliferation was observed after 24 h of exposure to 500 pg/mL of glucagon, possibly due to reaching a saturation point or the activation of inhibitory feedback mechanisms [43,49]. Regarding migration, α-TC1 cells showed a moderate increase in migration in the presence of 50 pg/mL of glucagon under high-glucose conditions (Figure 2N,P). This indicates that physiological levels of glucagon, paired with glucose, might slightly increase migration, which fits with the increased proliferation rate observed under the same conditions. Glucagon dynamics have been described as having a great impact on normal α-cell proliferation [50,51]. It is possible that, in a normal pancreas, glucagon is a regulator of tissue repair and equilibrium [52,53] and proliferation and migration are important phenomena involved.

The metabolic remodelling prompted by glucagon and glucose in pNET cells is evident in the heat maps and PCA and PLS-DA analyses (Figure 3A–I). The amino acid profiles showed a clear impact of glucagon and glucose availability. Arginine and glutamine were exclusively detected in pNET cell lines under high-glucose conditions (Figure 3A,B). Since the metabolism of glutamine and arginine is deeply connected, since they can be interconverted to answer cell needs [54], this suggests that their metabolism is influenced by glucose availability. A synergistic effect between glucose and glucagon in BON-1 cells indicates that glucagon also influences amino acid dynamics (Figure 4A). Conversely, the production of arginine and glutamine in QGP-1 cells seemed to be glucagon-independent (Figure 3B). Furthermore, alanine, glutamine, and lactate levels decreased upon glucose scarcity, which may be related to the diversion of these compounds to gluconeogenesis. Conversely, alanine, pyroglutamate, and serine increased in BON-1 and QGP-1 under high-glucose conditions (Figure 3A,B). Alanine holds a central role in glucose metabolism as a substrate for gluconeogenesis, at the cellular level, and systemically, as a player in the glucose-alanine cycle [7]. Since pyroglutamate results from the degradation of glutathione [55], its elevated levels in high-glucose conditions might indicate an increased demand for glutathione and consequently higher turnover, possibly as a way of maintaining the metabolic flow by sustaining redox reactions [56]. Accordingly, glycine levels were increased in high-glucose conditions for QGP-1 cells (Figure 3B), and glycine is a component of glutathione [7]. Serine is pivotal in several pathways like one-carbon metabolism and protein, purine, and pyrimidine synthesis [7]. Elevated serine levels in high-glucose contexts point to intensified serine synthesis driven by glucose availability. The increase in serine may also imply amplified needs in nucleotide synthesis, to sustain rapid proliferation and survival [7]. Proline metabolism by proline oxidase promotes the production of ROS and controls cell survival by inducing apoptosis [57]. The increased levels of proline in the extracellular media may contribute to increased cell viability since the import of proline is possibly reduced in glucose conditions. Contrarily, several amino acids, including isoleucine, lysine, methionine, phenylalanine, threonine, tryptophan, and valine, showed decreased levels in high-glucose conditions in QGP-1 cells (Figure 3B). These amino acids are nutritionally indispensable [58], so their reduced levels in high-glucose conditions might indicate an increased uptake to further supply metabolic pathways. The consistent observation that most amino acids levels were influenced by glucose conditions, irrespective of glucagon exposure, underscores the dominant role of glucose in dictating the metabolic behaviour of pNET cells, mainly QGP-1 cells.

Regarding organic acids, the increased levels of pyruvate in QGP-1 cells cultured in no-glucose conditions (Figure 3D) suggest a shift in metabolic priorities, with the activation of pathways also accounting for pyruvate production as fatty acids and cysteine catabolism [59]. Conversely, the increase in lactate levels in cells exposed to high glucose levels (Figure 3D,E) suggests an enhanced glycolytic activity. In QGP-1 cells, the contribution of glucagon to this increment might indicate a glucagon-mediated enhancement of glycolysis (Figure 3E). Accordingly, a glucagon-dependent hyperglycaemia is described as promoting cancer progression and angiogenesis under the activation of HIF1α pathways, which is the main controller of glycolysis [60]. In both BON-1 and QGP-1, the higher levels of isobutyrate in cells cultured in no-glucose than in glucose conditions (Figure 3A,D) demonstrates an adaptation to glucose scarcity, involving the activation of fatty acid or amino acid catabolism [61].

Nucleotide and nitrogen base metabolism are crucial to sustain cell proliferation, and the detection of hypoxanthine in BON-1 cells (Figure 3A), and the decreased levels of methylxanthine in all cell lines (Figure 3G–I) are in agreement with the proliferation results obtained in high-glucose conditions. Hypoxanthine is a purine derivative and is a precursor in the synthesis of nucleic acids [62] and a by-product of DNA repair mechanisms [62]. The decreased methylxanthine levels might indicate a reduced catabolism of purines or an altered adenosine metabolism [63].

In BON-1, glucagon increased the levels of tyramine in no-glucose conditions (Figure 3I and Figure S3G), indicating glucagon regulates catecholamine synthesis or secretion [64,65].

Regarding glucose itself, our results indicate that QGP-1 cells are the most efficient at consuming glucose, with an impressive 80% consumption rate (Figure 3L). In contrast, BON-1 cells consumed only 50% of the available glucose (Figure 3L). The differential consumption rates between these cell lines could be attributed to their distinct metabolic profiles and requirements, as demonstrated by their unique exometabolome. Regarding glucose production, BON-1 cells did not produce detectable levels of glucose, whereas QGP-1 cells evidently produced glucose (Figure 3J). This ability of QGP-1 cells to produce glucose, together with increased levels of alanine in high-glucose conditions, indicates gluconeogenesis is activated in these cells.

Non-malignant α-TC1 cells presented a metabolic profile different in some aspects from pNET cell lines. The increased levels of arginine in high-glucose conditions (Figure 3G), suggest an increased synthesis or decreased uptake, possibly driven by the availability of glucose. The observed decrease in lysine levels in high-glucose conditions (Figure 3G) indicates either increased uptake or catabolism, reflecting adaptive mechanisms in response to glucose availability. The increased levels of isobutyrate and succinate in high-glucose conditions (Figure S3F), suggest a decrease in TCA cycle activity [7,65], accounting for an increased export of these intermediates. The decreased levels of acetate, lactate, and pyruvate in high-glucose conditions (Figure S3F) indicate a reduced glycolytic activity or enhanced consumption of these intermediates in oxidative phosphorylation. The absence of succinate detection in the presence of glucagon and high glucose levels (Figure S3F) indicates that the TCA cycle is active, and cells are taking advantage of glucose for energetic purposes [7,66]. In α-TC1 cells, glucagon decreased the levels of tyramine in no-glucose conditions (Figure 3I and Figure S3G), indicating it impacts catecholamine metabolism [64,65]. Interestingly, α-TC1 cells consumed 30% of the available glucose, and glucagon increased glucose consumption by about 10% (Figure 3L). This suggests an autocrine regulatory role of glucagon in modulating glucose metabolism in α-cells. The observed increase in alanine levels in high-glucose conditions (Figure 3C) aligns with its role in gluconeogenesis, where alanine serves as a substrate for glucose production [67]. Importantly, α-TC1 cells were the most efficient glucose producers, with glucagon stimulating a 2-fold increase in production (Figure 3J). Such an observation is one of the most significant results of our study, underscoring the importance of α-cells in physiological contexts. Pancreatic neuroendocrine α-cells produce glucagon, which, in addition to its systemic role, acts on α-cells in an autocrine fashion to regulate their metabolism. This includes the activation of gluconeogenesis, thereby helping to resolve hypoglycaemia [48].

Overall, the NMR exometabolome analysis reveals that, in glucose-rich conditions, pNET cells predominantly utilize glycolysis, leading to increased lactate and alanine production. However, under glucagon stimulation, especially under glucose deprivation, pNET cells shift towards gluconeogenesis and amino acid metabolism, using these pathways to sustain their energy needs.

**Figure 6:**
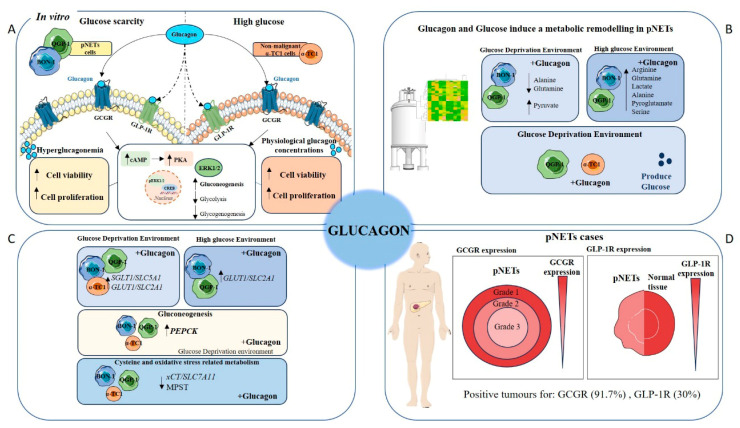
Glucagon has an intricate role in pNET cell survival and tumour progression. (**A**) Noteworthy is glucagon’s capacity to interact with both the GCGR and GLP-1R, introducing complexities to its role in cancer biology and potentially subverting disease-favouring mechanisms. Our findings confirm heightened GCGR expression in pNET cell lines (BON-1 and QGP-1) under glucose deprivation, especially in hyperglucagonemic conditions. Additionally, we observe significant GCGR expression in non-malignant pancreatic α-TC1 cells under normal physiological glucagon concentrations. This study uncovers the influence of glucagon on the MAPK pathway, evidenced by increased pERK1/2 levels in BON-1 and QGP-1 cells under glucagon stimulation during glucose deprivation. Examining the impact of glucagon on cell features, certain malignant cells, such as BON-1, which does not produce glucose, exhibit increased metabolic viability and proliferation in glucose-deprived conditions under hyperglucagonemia-compatible concentrations. (**B**) Metabolic remodelling, explored via NMR spectroscopy, reveals increased levels of arginine, glutamine, lactate, alanine, pyroglutamate, and serine in pNET cell lines under high-glucose conditions. In glucose scarcity, alanine and glutamine decrease, potentially diverting to gluconeogenesis, while pyruvate increases. BON-1 cells do not produce glucose, but interestingly, QGP-1 cells and α-TC1 cells do, further enhanced by glucagon in α-TC1 cells. (**C**) Genetic mechanisms and metabolic shifts in pNETs are comprehensively explored, highlighting increased *SGLT1/SLC5A1* and *GLUT1/SLC2A1* expression in response to glucagon under glucose deprivation. Investigating gluconeogenesis, the findings highlight its significance in pNET cells, especially concerning the influence of glucagon, under which PEPCK expression is increased. Cysteine and oxidative stress-related metabolism reveal downregulation of *xCT/SLC7A11* and MPST expression in both BON-1 and QGP-1 cells under glucagon’s influence, independent of glucose conditions. (**D**) Investigating GCGR expression in pNETs uncovers potential therapeutic insights, with an observed inverse association between GCGR intensity staining and tumour grade. Despite grade 3 tumour variability, GCGR positivity in pNETs implies a biological role. Reduced GLP-1R expression in pNETs, in contrast to normal tissue, indicates modified glucagon functioning patterns that favour GCGR. Among the 60 cases, 91.7% (55/60) were GCGR-positive, while 30% (18/60) were GLP-1R-positive. This comprehensive investigation deepens our comprehension of the multifaceted role of glucagon in pNETs, providing insights for potential targeted therapeutic interventions.

In the complex landscape of pNETs, understanding the genetic mechanisms and regulation underlying the foremost metabolic shifts offers a more comprehensive view (Figure 6). In deprived glucose conditions, both BON-1 (Figure 4A) and QGP-1 (Figure 4C) increased the expression of genes encoding the glucose transporters *GLUT1/SLC2A1* and *SGLT1/SLC5A1*, which may constitute a compensatory mechanism. In high-glucose conditions, a contrasting behaviour was observed in pNET cells. BON-1 (Figure 4B) showed an increased expression of *GLUT1/SLC2A1* and *SGLT1/SLC5A1*, while in QGP-1 (Figure 4D) the expression of both genes decreased. This shows a decreased reliance on glucose supplementation in QGP-1 compared to BON-1 cells, which fits with QGP-1 cells’ efficiency in producing glucose.

Exploring gluconeogenesis, both BON-1 and QGP-1 cells, upon glucose scarcity, upregulated *PEPCK* gene expression in response to glucagon stimulus (Figure 4A,C), aligning with literature that assigns *PEPCK* as a pivotal enzyme in pNETs [68,69,70]. Considering cysteine and oxidative stress-related metabolism, BON-1 and QGP-1 cells decreased *xCT/SLC7A11* expression in response to glucagon, independent of glucose availability (Figure 4A–D). In BON-1 (Figure 4A), this corresponds with the decreased expression of *MPST*, encoding an enzyme central to cysteine catabolism with the consequent production of pyruvate [71], which fits with the decreased levels of pyruvate in no-glucose conditions. These results indicate glucagon promotes adjustments in redox control metabolism, given the role of *xCT/SLC7A11* in the uptake of cysteine for glutathione synthesis [7,71], which is reinforced by the decreased *GSS* expression in response to glucagon exposure in BON-1 and QGP-1 cells.

In the context of pNET pathology, the expression of the GCGR presents a promising pathophysiological marker or therapeutic target (Figure 6). Our study found that GCGR expression was inversely associated with tumour grade (Figure 5A,B), suggesting possible differences in glucagon signalling between early-stage and more advanced tumours. Therefore, further investigations with a larger sample size and more grade 3 tumours will clarify this correlation. Nonetheless, 91.7% of cases were positive for GCGR expression suggesting a biological role for GCGR in disease that deserves to be investigated (Table 4). Another interesting observation is the decreased expression of GLP-1R in pNETs compared to normal tissue (Table 3 and Figure 5C), which may reflect a disrupted insulin–glucagon axis. This is supported by the negative correlation between the two receptors (Table 4) and the fact that 92.8% of GLP-1R-negative cases are positive for GCGR expression. Considering the role of GLP-1R in glucose homeostasis [29,72,73], its diminished expression in pNETs could indicate a dysregulation in the insulin–glucagon axis [28]. This is reinforced by the maintenance of high levels of GLP-1R in insulinomas (Figure 5E), highlighting again the need for a systematic functional characterization of pNETs. Our in vitro analyses further corroborate the observed differences in GLP-1R expression between pNETs and healthy tissue (Figure 5F–L); nevertheless, pNET cell lines used in this study are not insulinoma-derived, and the lack of pNET representative cell lines is a technical difficulty. Clinically, this inverse relationship could serve as a prognostic indicator, as high GCGR and low GLP-1R expression may signal more aggressive pNET phenotypes. Future studies could investigate the prognosis and therapeutic power of GCGR/GLP-1R ratio in pNETs.

## 5. Conclusions

Pancreatic neuroendocrine tumours (pNETs) present significant diagnostic challenges due to their often asymptomatic nature, underscoring the urgent need for deeper biological insights to identify novel biomarkers and therapeutic targets. Our study highlights glucagon’s pivotal role as a metabolic regulator in pNET biology, demonstrating that its interaction with the GCGR and GLP-1R orchestrates complex adaptive responses, particularly under conditions of glucose deprivation.

The differential responses of BON-1, QGP-1, and α-TC1 cells to glucagon highlight the complex and heterogeneous nature of pNET biology, as well as the physiological specificity of glucagon action in normal α-cells. pNETs present significant diagnostic challenges due to their often asymptomatic nature, emphasizing the urgent need for a deeper understanding of their underlying biology to identify novel biomarkers and therapeutic targets. Our study demonstrates that glucagon, through its interaction with GCGR and/or GLP-1R, plays a pivotal role in orchestrating metabolic and adaptive responses in both tumour and normal endocrine cells, particularly under conditions of glucose deprivation.

In BON-1 cells, glucagon significantly enhanced metabolic viability and proliferation under glucose-deprived conditions, suggesting a strong reliance on glucagon-triggered survival pathways. This effect is likely due to BON-1’s limited ability to produce glucose endogenously, necessitating external signals to sustain cell survival. The upregulation of GCGR expression and activation of the MAPK/ERK pathway further support glucagon’s role as a compensatory mitogenic and pro-survival factor in this context. These findings point to a glucagon-mediated metabolic remodelling in BON-1 cells, confirmed by NMR and involving the activation of pathways related to gluconeogenesis and amino acid utilization.

In contrast, QGP-1 cells exhibited a more restrained response to glucagon. While a modest rescue in cell viability was observed after 48 h of glucagon exposure, no significant proliferation increase occurred under glucose-deprived conditions. This muted response appears to stem from QGP-1’s intrinsic capacity for glucose production, allowing the cells to adapt more efficiently to nutrient scarcity without heavily relying on glucagon signalling. These contrasting behaviours between BON-1 and QGP-1 cells reflect the metabolic heterogeneity within pNETs and underscore how distinct adaptive strategies may influence their response to hormonal stimuli and potentially to therapeutic interventions.

The α-TC1 cell line, representative of normal pancreatic α-cells, displayed a more physiologically predictable response to glucagon. In high-glucose conditions, glucagon stimulated both viability and proliferation, in line with its known role in supporting α-cell function. Under glucose-deprived conditions, α-TC1 cells initially showed reduced viability, but a significant recovery was observed at 48 h, indicating an adaptive pro-survival response, likely tied to endogenous glucose production mechanisms. Additionally, α-TC1 cells exhibited a moderate increase in migration in response to glucagon, aligning with their proliferative behaviour and suggesting a potential role for glucagon in islet tissue maintenance and repair.

Together, these findings reveal that glucagon promotes cell viability, proliferation, and migration in a cell type- and context-dependent manner. In pNET cells, glucagon’s effects are closely tied to each cell line’s metabolic capabilities and receptor expression profiles. Clinically, the high prevalence of GCGR expression in pNET tissues and its inverse correlation with tumour grade, alongside the reduced GLP-1R expression, point to disrupted insulin–glucagon signalling in tumour progression. This receptor expression profile holds promise as a prognostic marker and potential therapeutic target.

In summary, this study expands our understanding of glucagon’s role in pNET pathophysiology, emphasizing the significance of metabolic regulation in tumour adaptation and highlighting GCGR as a key candidate for future biomarker development and targeted therapy. Further research into the insulin–glucagon axis and metabolic signalling pathways could pave the way for improved diagnostic and treatment strategies in pNET management.

## Figures and Tables

**Table 1:** Primers sequences used to assess the quantification of listed key human metabolic genes. The gene encoding hypoxanthine phosphoribosyltransferase-1 (*HPRT*) was used as the housekeeping gene.

Protein	Gene	Primer Forward (5′-3′)	Primer Reverse (5′-3′)
3-mercaptopyruvate sulfurtransferase	*MPST*	CTTCATCAAGACCTACGAGGAC	GGTAGTGGCCAGGTTCAATG
cystine/glutamate antiporter (xCT)	*SLC7A11*	GGTCCTGTCACTATTTGGAGC	GAGGAGTTCCACCCAGACTC
monocarboxylate transporter 1	*MCT1*	GCTGGGCAGTGGTAATTGGA	CAGTAATTGATTTGGGAAATGCAT
monocarboxylate transporter 3	*MCT4*	CACAAGTTCTCCAGTGCCATTG	CGCATCCAGGAGTTTGCCTC
lactate dehydrogenase A	*LDHA*	CTTGCTCTTGTTGATGTCATC	CAGCCGTGATAATGACCAGC
lactate dehydrogenase B	*LDHB*	GAGCCTTCTCTCTCCTGTG	CTGATAGCACACGCCATACC
glucose transporter 1	*GLUT1*	CACGGCCTTCACTGTCGTG	GGACATCCAGGGTAGCTGC
glucose-6-phosphate dehydrogenase	*G6PD*	GGCAACAGATACAAGAACGTGAAG	GCAGAAGACGTCCAGGATGAG
sodium-coupled glucose transporter 1	*SGLT1*	CACAGATCAGGTCATTGTGC	CATGATGAACATGGGCATCAGC
phosphoenolpyruvate carboxykinase	*PEPCK*	GCGGATCATGACGCGGATG	GAGCGTCAGCTCCGGGTTG
6-phosphofructo-2-kinase/fructose-2,6-biphosphatase 1	*PFKFB1*	CCAGTATCGACGAGAGGCAG	CTCTGGTAGTGTTGGTGGCATC
glutathione synthetase	*GSS*	GAGAGAGGGTGGAGGTAAC	CCATGAGGATGTAGGAGGCC
hypoxanthine phosphoribosyltransferase-1	*HPRT*	TGACACTGGCAAAACAATGCA	GGTCGTTTTTCACCAGCAAGCT

**Table 2:** Correlation between GCGR/GLP1R expression in pNETs with biological parameters, tumour staging, and immunohistochemistry (IHC) staining score (0—absent, 1—weak, 2—moderate, and 3—strong) for GCGR and GLP1 and metastasis.

	GCGR			GLP1R		
Variables	Negative (%)	Positive (%)	*p* Value	Negative (%)	Positive (%)	*p* Value
All patients	5 (8.3)	55 (91.7)		42 (70.0)	18 (30.0)	
Gender			0.023 *			0.014 *
Males (38.3%)	0 (0)	23 (100)		20 (87.0)	3 (13.0)	
Females (61.7%)	5 (13.5)	32 (86.5)		22 (59.5)	15 (40.5)	
Age (24–85 years)			0.647			0.581
≤60	2 (6.7)	28 (93.3)		22 (73.3)	8 (26.7)	
≥60	3 (10.0)	27 (90.0)		20 (66.7)	10 (33.3)	
Tumour size (cm)			0.256			0.076
≤2 cm	3 (15.8)	16 (84.2)		10 (52.6)	9 (47.3)	
≥2 cm	2 (5.0)	38 (95.0)		31 (77.5)	9 (22.5)	
Tumour grade			0.349			0.652
Grade 1	4 (11.1)	32 (88.8)		26 (72.2)	10 (27.8)	
Grade 2/3	1 (4.2)	23 (95.8)		16 (66.7)	8 (33.3)	
Tumour staging			0.755			0.904
T1/T2	3 (7.5)	37 (92.5)		28 (70.0)	12 (30.0)	
T3/T4	1 (5.3)	18 (94.7)		13 (68.4)	6 (31.6)	
Lymph node metastasis			0.952			0.635
NX/N0	3 (6.67)	42 (93.3)		32 (71.1)	13 (28.9)	
N1	1 (7.1)	13 (92.9)		9 (64.3)	5 (35.7)	
Distant metastasis			0.790			0.512
MX/M0	4 (6.9)	54 (93.1)		40 (69.0)	18 (31.0)	
M1	0 (0)	1 (100.0)		1 (100.0)	0 (0)	
Intensity staining score			0.023 *			0.652
0–1	5 (13.9)	31 (86.1)		26 (72.2)	10 (27.8)	
2–3	0 (0)	24 (100.0)		16 (66.7)	8 (33.3)	

* Statistically significant at *p* < 0.05.

**Table 3:** Association between GCGR/GLP1R intensity staining score (0—absent, 1—weak, 2—moderate, and 3—strong) with pNET/normal pancreatic tissue.

	GCGR Intensity Score			GLP1R Intensity Score		
Variables	0–1 (%)	2–3 (%)	*p* Value	0–1 (%)	2–3 (%)	*p* Value
Group						
pNET	36 (60.0)	24 (40.0)	0.102	48 (80.0)	12 (20.0)	<0.001 *
Normal pancreatic tissue	27 (45.0)	33 (55.0)		29 (48.3)	31 (51.7)	

* Statistically significant at *p* < 0.05.

**Table 4:** Association of GCGR and GLP-1R expression in pNETs and the correlation between the expression of the two receptors.

pNET Total Cases (N = 60)			
	Positive Cases (%)	Negative Cases (%)	*p* Value
GCGR	55 (91.7)	5 (8.3)	1.039 × 10^−11^ *
GLP-1R	18 (30)	42 (70)	0.003
**GLP-1R-negative cases (N = 42)**			
	**Cases (%)**		***p *** **value**
GCGR positive	39 (92.9)		5.3616 × 10^−9^ *
GCGR negative	3 (7.1)		

* Binomial exact test used, statistically significant at *p* < 0.05.

## Data Availability

This study is available at the NIH Common Fund’s National Metabolomics Data Repository (NMDR) website, the Metabolomics Workbench, https://www.metabolomicsworkbench.org, where it has been assigned Study ID ST003584. The data can be accessed directly via the project DOI: http://dx.doi.org/10.21228/M8FF9T.

## Supplementary Materials

The following supporting information can be downloaded at: https://www.mdpi.com/article/10.3390/molecules30132736/s1, Figure S1: Representative images of immunohistochemistry (IHC) intensity score.; Figure S2: Permutation test cross validation of PLS-DA scores plot.; Figure S3: Glucagon impact differently BON-1, QGP-1 and α-TC1 cells, pNETs cell line consume more glucose than noncancer α-TC1 cells, which is additionally an efficient producer of glucose stimulated by glucagon.

## Abbreviations

pNETs	pancreatic neuroendocrine tumours
TME	tumour microenvironment
TOME	tumour and organ microenvironment
GPCR	G-protein coupled receptor
GCGR	glucagon receptor
GLP-1R	glucagon-like peptide-1 receptor
OXPHOS	oxidative phosphorylation
MAPK	mitogen-activated protein kinase
ERK	extracellular signal-regulated kinase
cAMP	second messenger cyclic AMP
PKA	protein kinase A
ACH	α-cell hyperplasia
*MPST*	3-mercaptopyruvate sulfurtransferase
*SLC7A11*	cystine/glutamate antiporter (xCT)
*MCT1*	monocarboxylate transporter 1
*MCT4*	monocarboxylate transporter 4
*LDHA*	lactate dehydrogenase A
*LDHB*	lactate dehydrogenase B
*GLUT1*	glucose transporter 1
*G6PD*	glucose-6-phosphate dehydrogenase
*SGLT1*	sodium-coupled glucose transporter 1
*PEPCK*	phosphoenolpyruvate carboxykinase
*PFKFB1*	6-phosphofructo-2-kinase/fructose-2,6-biphosphatase 1
GSS	glutathione synthetase
HPRT	hypoxanthine phosphoribosyltransferase-1
