# Supplementary material for: Glucagon and Glucose Availability Influence Metabolic Heterogeneity and Malignancy in Pancreatic Neuroendocrine Tumour (pNET) Cells: Novel Routes for Therapeutic Targeting
Source: Molecules. 2025 Jun 25;30(13):2736. doi: 10.3390/molecules30132736 (PMC12251001; doi:10.3390/molecules30132736)
Supplement: Supplementary file 1 [file molecules-30-02736-s001.zip › molecules-3658111-supplementary.pdf]

Supplementary Figures

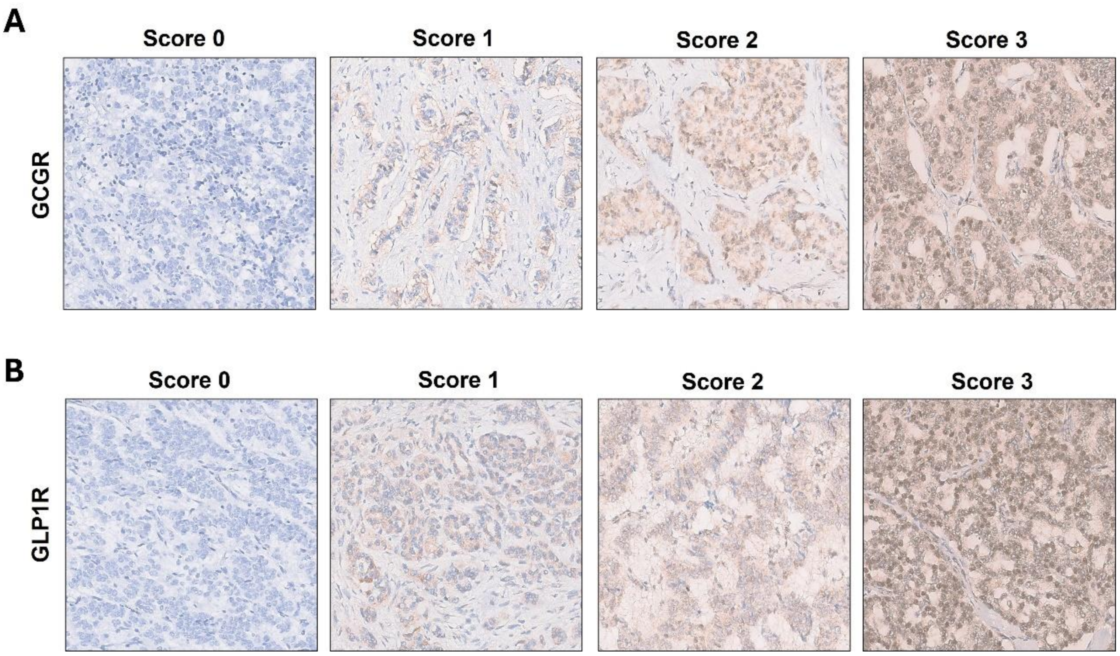

Supplementary Figure S1: Representative images of immunohistochemistry (IHC) intensity score.

Representative images of IHC intensity score (0-absent, 1- weak, 2- moderate and 3-strong) are presented for A) GCGR and B) GLP1-R detection.

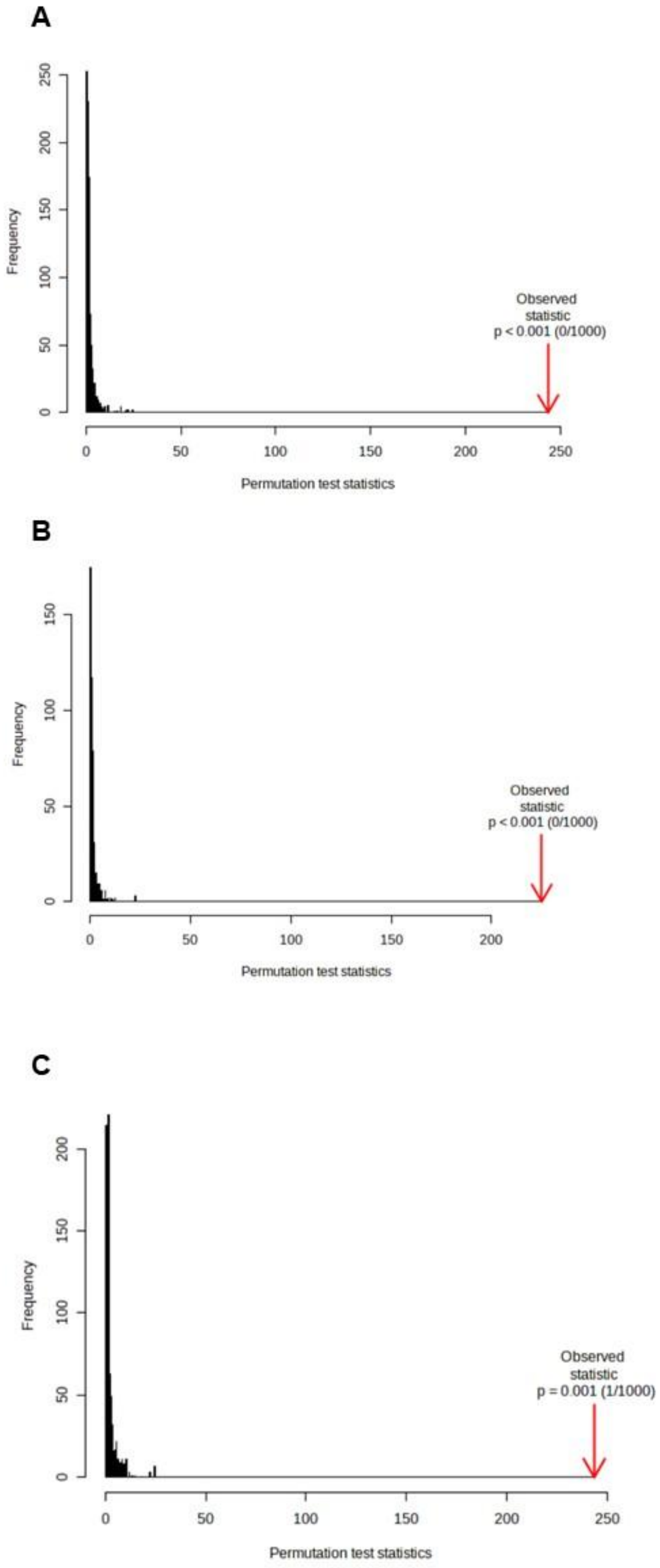
A
Frequency
Observed
statistic
p < 0.001 (0/1000)
Permutation test statistics
B
Frequency
Observed
statistic
p < 0.001 (0/1000)
Permutation test statistics
C
Frequency
Observed
statistic
p = 0.001 (1/1000)
Permutation test statistics

Supplementary Figure S2: Permutation test cross validation of PLS-DA scores plot.

This supplementary figure illustrates the robustness of our predictive model through a permutation test-based cross-validation approach with 1000 permutations. ABC), presents the results for different cell lines, BON-1, QGP-1, and α-TC1 cell lines, respectively.

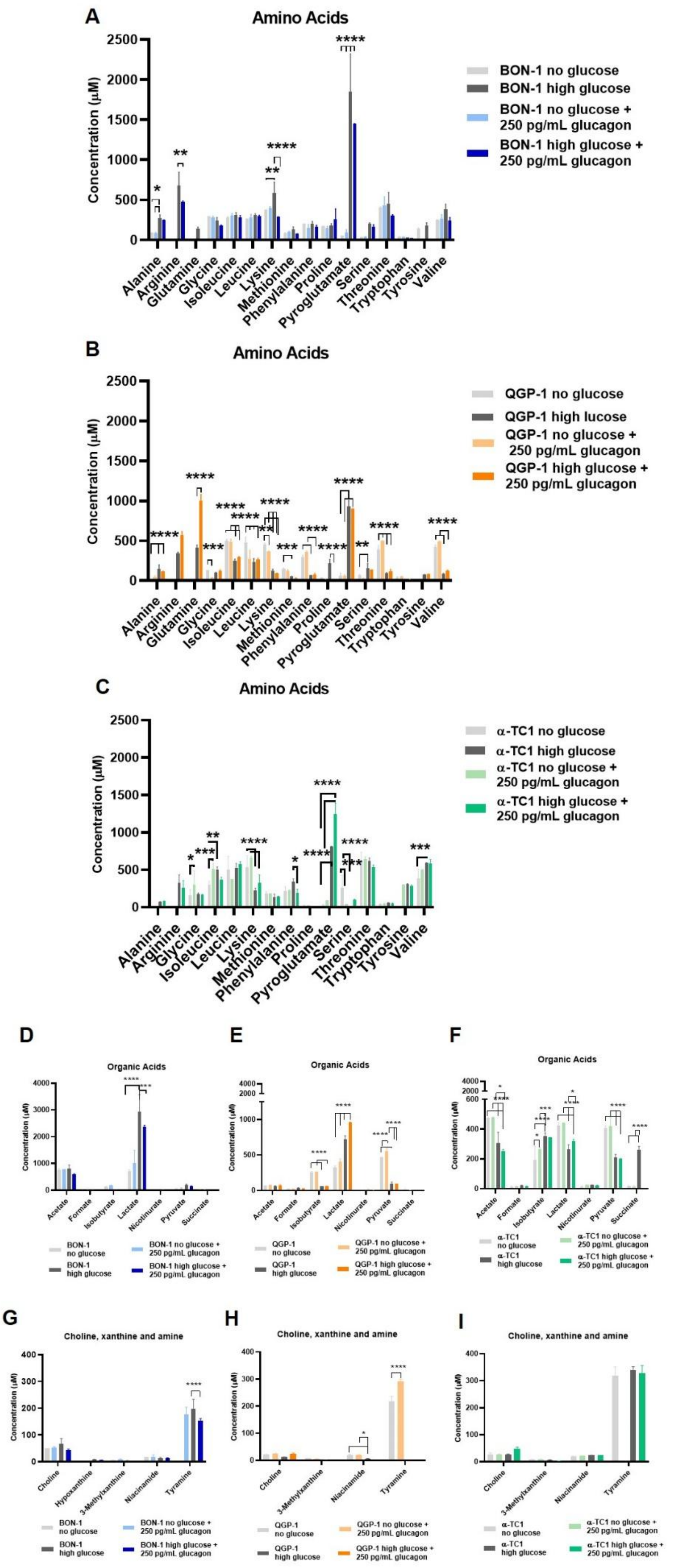
A
Amino Acids
Concentration (µM)
BON-1 no glucose
BON-1 high glucose
BON-1 no glucose + 250 pg/mL glucagon
BON-1 high glucose + 250 pg/mL glucagon
Alanine
Arginine
Glutamine
Glycine
Isoleucine
Leucine
Lysine
Methionine
Phenylalanine
Proline
Pyroglutamate
Serine
Threonine
Tryptophan
Tyrosine
Valine
B
Amino Acids
Concentration (µM)
QGP-1 no glucose
QGP-1 high lucose
QGP-1 no glucose + 250 pg/mL glucagon
QGP-1 high glucose + 250 pg/mL glucagon
C
Amino Acids
Concentration (µM)
α-TC1 no glucose
α-TC1 high glucose
α-TC1 no glucose + 250 pg/mL glucagon
α-TC1 high glucose + 250 pg/mL glucagon
D
Organic Acids
E
Organic Acids
F
Organic Acids
Acetate
Formate
Isobutyrate
Lactate
Nicotinurate
Pyruvate
Succinate
G
Choline, xanthine and amine
H
Choline, xanthine and amine
I
Choline, xanthine and amine
Choline
Hypoxanthine
3-Methylxanthine
Niacinamide
Tyramine

Supplementary Figure S3: Glucagon impact differently BON-1, QGP-1 and α-TC1 cells, pNETs cell line consume more glucose than noncancer α-TC1 cells, which is additionally an efficient producer of glucose stimulated by glucagon.

Identified and quantified amino acids in (A) BON-1, (B) QGP-1 and (C) α-TC1 cell line. Identified and quantified organic acids in (D) BON-1, (E) QGP-1 and (F) α-TC1 cell line, respectively. Identified and quantified choline, xanthine and amines in (G) BON-1, (H) QGP-1 and (I) α-TC1 cell line, respectively. Statistical analysis was performed using ONE-way ANOVA. *$p<0.05$; **$p<0.01$; ***$p<0.001$, indicate a significant difference between each mean of each group.
